# Direct Measurements of Covalently Bonded Sulfuric Anhydrides from Gas-Phase Reactions of SO_3_ with Acids under Ambient Conditions

**DOI:** 10.1021/jacs.4c04531

**Published:** 2024-05-21

**Authors:** Avinash Kumar, Siddharth Iyer, Shawon Barua, James Brean, Emin Besic, Prasenjit Seal, Manuel Dall’Osto, David C. S. Beddows, Nina Sarnela, Tuija Jokinen, Mikko Sipilä, Roy M. Harrison, Matti Rissanen

**Affiliations:** † Aerosol Physics Laboratory, Physics Unit, Faculty of Engineering and Natural Sciences, Tampere University, 33720 Tampere, Finland; ‡ School of Geography, Earth & Environmental Sciences, University of Birmingham, Birmingham B15 2TT, United Kingdom; § Institute of Marine Science, Consejo Superior de Investigaciones Científicas (CSIC), Barcelona 08003, Spain; ∥ National Centre for Atmospheric Science, School of Geography, Earth and Environmental Sciences, University of Birmingham, Edgbaston, Birmingham B15 2TT, U.K.; ⊥ Institute for Atmospheric and Earth System Research (INAR)/Physics, Faculty of Science, University of Helsinki, P.O. Box 64, Helsinki 00014, Finland; # Climate & Atmosphere Research Centre (CARE-C), The Cyprus Institute, P.O. Box 27456, Nicosia 1645, Cyprus; ∇ Department of Chemistry, University of Helsinki, P.O. Box 55, 00014 Helsinki, Finland

## Abstract

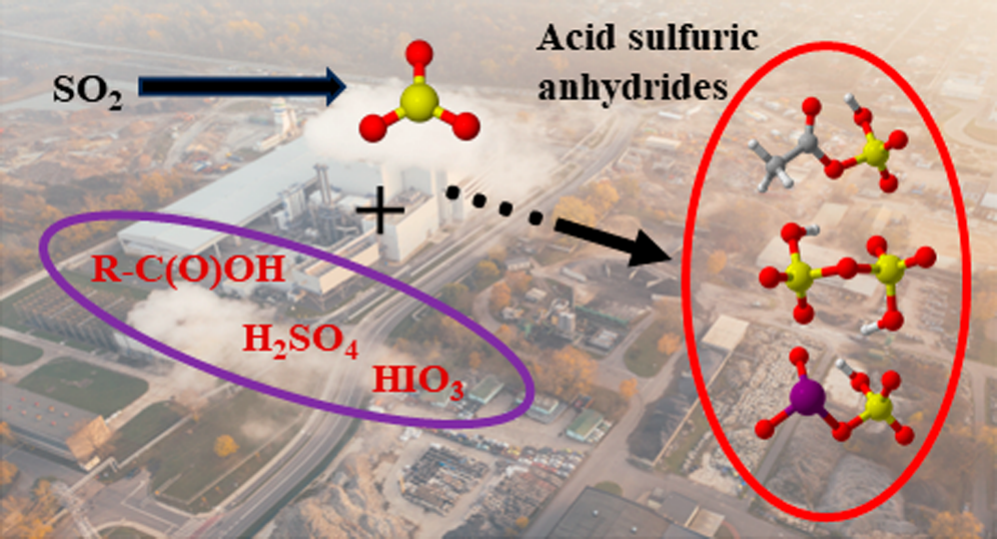

Sulfur trioxide (SO_3_) is an important oxide of sulfur and a key intermediate in the formation of sulfuric acid (H_2_SO_4_, SA) in the Earth’s atmosphere. This conversion to SA occurs rapidly due to the reaction of SO_3_ with a water dimer. However, gas-phase SO_3_ has been measured directly at concentrations that are comparable to that of SA under polluted mega-city conditions, indicating gaps in our current understanding of the sources and fates of SO_3_. Its reaction with atmospheric acids could be one such fate that can have significant implications for atmospheric chemistry. In the present investigation, laboratory experiments were conducted in a flow reactor to generate a range of previously uncharacterized condensable sulfur-containing reaction products by reacting SO_3_ with a set of atmospherically relevant inorganic and organic acids at room temperature and atmospheric pressure. Specifically, key inorganic acids known to be responsible for most ambient new particle formation events, iodic acid (HIO_3_, IA) and SA, are observed to react promptly with SO_3_ to form iodic sulfuric anhydride (IO_3_SO_3_H, ISA) and disulfuric acid (H_2_S_2_O_7_, DSA). Carboxylic sulfuric anhydrides (CSAs) were observed to form by the reaction of SO_3_ with C_2_ and C_3_ monocarboxylic (acetic and propanoic acid) and dicarboxylic (oxalic and malonic acid)–carboxylic acids. The formed products were detected by a nitrate-ion-based chemical ionization atmospheric pressure interface time-of-flight mass spectrometer (NO_3_^–^-CI-APi-TOF; NO_3_^–^-CIMS). Quantum chemical methods were used to compute the relevant SO_3_ reaction rate coefficients, probe the reaction mechanisms, and model the ionization chemistry inherent in the detection of the products by NO_3_^–^-CIMS. Additionally, we use NO_3_^–^-CIMS ambient data to report that significant concentrations of SO_3_ and its acid anhydride reaction products are present under polluted, marine and polar, and volcanic plume conditions. Considering that these regions are rich in the acid precursors studied here, the reported reactions need to be accounted for in the modeling of atmospheric new particle formation.

## 1 Introduction

Sulfur oxides (SO_*x*_) are major air pollutants that contribute to acid rain and particulate matter formation in the Earth’s atmosphere.^1−3^ Among the SO_*x*_, sulfur dioxide (SO_2_) is the most emitted, with burning of fuels and human activities being the dominant source along with natural emission processes.^4−6^ Sulfur trioxide (SO_3_) is formed by the gas-phase oxidation of SO_2_ and is an important intermediate toward the formation of sulfuric acid (SA, H_2_SO_4_), which plays a key role in atmospheric new particle formation (NPF).^7−10^ These oxides of sulfur can also react on organic surfaces generating organo-sulfates (OS), which affect the physicochemical properties of aerosol particles.^11−16^ The aerosol particles in the atmosphere are well-known to influence climate, air quality, and human health.^17−22^ Numerous field measurements have shown that OS are present in atmospheric particles in concentrations high enough to affect atmospheric physicochemical processes.^13,23−25^ It has been estimated that OS contribute to up to 30 and 12% of the organic mass and total sulfur, respectively, at a forest site in Hungary.^26,27^

Carboxylic acids comprise a major fraction of the organics present in the atmosphere^17,27,28^ and are largely sourced from photo-oxidation of vehicle emissions and biomass burning. They can also be directly emitted by vegetation and from cooking.^17,28−34^ They are observed both in the gas and particle phases and are known to stabilize the prenucleation clusters by binding strongly with other species in the atmosphere.^35−40^ The concentration of acetic acid was measured to be in the range of 1.5–8.4 × 10^10^ molecules cm^–3^ in northwestern USA and 0.5–43.8 × 10^10^ molecules cm^–3^ at an urban site in California.^41,42^ Among dicarboxylic acids, oxalic acid is the most prevalent, with concentrations ranging from 10^7^ to 10^9^ molecules cm^–3^ in urban and nonurban atmospheres.^43−46^ In certain remote locations, carboxylic acids can account for as much as 80–90% of acidity in the precipitation.^47,48^ They can also contribute to the formation of cloud condensation nuclei.^49,50^ SA and iodic acid (IA) are well-known drivers of tropospheric nucleation processes.^8,10,51−65^ Daytime SA concentrations are estimated to be in the range of 10^6^–10^7^ molecules cm^–3^ or below in most urban and remote locations and it can even reach up to 10^8^ molecules cm^–3^ in polluted environments.^66−69^ The concentration of IA in the polluted urban environment of Beijing and Nanjing reaches up to 10^6^ molecules cm^–3^ in the summer months.^70^ The IA concentration sourced by biogenic emissions near coastal environments can even reach up to 10^8^ cm^–3^.^59,71,72^

Only a few studies have investigated the gas-phase formation mechanism of organosulfur compounds from the reactions of SO_3_ with organics present in the atmosphere. As the atmospheric lifetime of gaseous SO_3_ is traditionally thought to be extremely short due to its fast reaction with the water dimer (to produce SA), its reaction with other atmospherically relevant species is not well studied. However, Yao et al.^73^ recently measured appreciable levels of SO_3_ during the winter (∼4 × 10^4^–1.9 × 10^6^ molecules cm^–3^) and summer months (∼5 × 10^3^–1.4 × 10^5^ molecules cm^–3^) in urban Beijing, using a nitrate-chemical ionization atmospheric pressure interface-long-time-of-flight (nitrate-CI-APi-LTOF) mass spectrometer. This indicates that significant steady-state concentrations of gaseous SO_3_ could exist under the polluted conditions of mega-cities.

Mackenzie et al.^74^ performed laboratory experiments and theoretical calculations to study the reaction of formic acid with SO_3_ under supersonic jet conditions, with the products characterized by Fourier transform microwave (FTMW) spectroscopy. They reported that the reaction produces a covalently bonded formic sulfuric anhydride (FSA) product formed by the cycloaddition of SO_3_ to formic acid. Subsequent experimental studies from the same laboratory have similarly used FTMW spectroscopy to report the formation of carboxylic sulfuric anhydrides (CSAs) from the reaction of SO_3_ with acetic, acrylic, trifluoroacetic, propiolic, and pivalic acids.^75−79^ Liu et al.^80^ carried out computational calculations for the reaction of SO_3_ with methanol to form methyl hydrogen sulfate and reported it to be competitive with the formation process of SA in dry and polluted regions and can reduce SA concentration up to 87%. Li et al.^81^ studied the formation of sulfamic acid from the reaction of SO_3_ with ammonia (NH_3_) and reported that the energy barrier of SO_3_–NH_3_ reaction gets lowered upon NH_3_ acting as a self-catalyst. It is a competitive loss pathway for SO_3_ when compared to the conventional SO_3_–H_2_O system in dry and highly polluted regions with relatively higher concentrations of ammonia. Sarkar et al.^82^ also calculated the catalytic effect of water and NH_3_ on SO_3_–NH_3_ and SO_3_–H_2_O reaction systems. They reported that the NH_3_-catalyzed pathway is faster than the H_2_O-catalyzed pathway for both systems, and NH_3_-catalyzed ammonolysis of SO_3_ shows a higher rate coefficient than NH_3_-catalyzed hydrolysis reaction of SO_3_. Long et al.^83^ studied the reaction of nitric acid (HNO_3_) with SO_3_ to form HOSO_2_–NO_3_ as a product, which has implications for understanding the sulfur partitioning in the stratosphere. Their kinetic calculations indicate that the SO_3_–HNO_3_ reaction system is competitive with the SO_3_–H_2_O system and is two times higher than the HONO_2_–OH reaction system in the altitude range of 25–35 km. Some recent theoretical studies report that the reactions of SO_3_ with other acids such as dicarboxylic, tricarboxylic, and aromatic carboxylic acids are kinetically feasible and competitive.^84−89^ This variety of CSAs was found to form more stable clusters with atmospheric nucleation agents such as SA, NH_3_, and dimethyl amine compared to their respective acid precursors, which suggests it to be a potential participator in NPF.^84−89^ There is only one computational study that reports the formation of disulfuric acid from the reaction of SA with SO_3_ and its role in aerosol particle formation in the gas phase and air–water interface.^90^

To the best of our knowledge, there are only six experimental studies that report the formation of CSA from the reaction of SO_3_ with carboxylic acids, but none under the actual ambient temperature and pressure conditions of the real atmosphere.^74−79^ These experiments were performed under supersonic expansion where the samples were mixed at higher pressure (usually more than 1 atm), and characterization was performed in low-temperature and -pressure conditions.

In this work, we carried out the reaction of SO_3_ with monocarboxylic acids (acetic acid (AA) and propionic acid (PA)), dicarboxylic acids (oxalic acid (OA) and malonic acid (MA)), SA, and IA in a laminar flow tube at atmospheric pressure and room temperature and monitored the products using a chemical ionization atmospheric pressure interface time-of-flight mass spectrometer with nitrate reagent ion (NO_3_^–^-CI-APi-TOF, henceforth NO_3_^–^-CIMS). This study is the first experimental demonstration of the formation and detection of disulfuric acid (DSA) and iodic sulfuric anhydride (ISA) under atmospherically relevant conditions of temperature and pressure in the gas phase. Additionally, an identification of IO_2_SO_3_H (i.e., iodous acid sulfate, HIO_2_–SO_3_) was made during the experiments with the HIO_3_–SO_3_ system. This work is a combined experimental and computational approach to understanding the interaction of SO_3_ with several of the prevalent organic and inorganic species in the atmosphere.

## 2 Methodology

### 2.1 Experimental Setup

A NO_3_^–^-CIMS instrument was used to detect the formed CSAs, DSA, and ISA.^69,91^ The experiments were performed in a borosilicate glass flow tube reactor (length = 100 cm and i.d. = 5 cm) coupled to the NO_3_^–^-CIMS. All of the experiments were performed at a laminar flow condition at room temperature and 1 atm pressure using zero air as a bath gas. The residence time for the chemical species to react in the flow tube was estimated to be around 11 s. SO_3_ was produced in situ by the reaction of SO_2_ with OH radicals or stabilized Criegee intermediate (sCI) in the presence of oxygen. The OH radicals and sCI were produced by the reaction of tetramethylethylene (TME, C_6_H_12_) with ozone. The flows of the gases were set by means of calibrated mass flow controllers, which were further utilized to calculate the concentration of a chemical species in the reaction mixture. Water was added to the reaction system to convert the formed SO_3_ to SA, which shows that the latter plays no role in the formation of the sulfuric anhydride products. A more detailed explanation of the inlet flows, chemical system, concentration of the reactants, and the overall experimental setup is given in the Supporting Information (S1.1–S1.3).

### 2.2 Theoretical Methods

Systematic conformational sampling was carried out using the molecular mechanics Merck-Molecular-Force-Field (MMFF) methods with the Spartan ’18 and ’20 programs (Wavefunction, Inc.).^92−94^ The generated conformers were optimized using density functional theory (DFT) methods, first at the low B3LYP/6-31+G(d) level of theory and later at the more accurate ωB97X-D/aug-cc-pV(T+d)Z level of theory for conformers within 2 kcal/mol of the lowest conformer in relative electronic energies. The DFT calculations were carried out using the Gaussian 16 program.^95^ Transition-state (TS) geometries were found by optimizing guess structures with the bond distances corresponding to the TS constrained, followed by unconstrained TS optimizations. Once the correct TS geometry was found, a conformational sampling step was carried out with the TS-associated bond distances constrained, followed by constrained and TS optimizations. The energies of the lowest energy reactant, reaction complex, TS, and product structures were refined by recalculating their single-point electronic energies at the RHF-RCCSD(T)-F12a/VDZ-F12 level using the Molpro 2022.2.2 program.

Binding energies of the acid sulfuric anhydrides-NO_3_^–^ clusters were calculated by performing systematic conformer sampling of the acid sulfuric anhydride molecule, its associated deprotonated form, and the cluster. Identical steps of low-level and higher-level DFT geometry optimizations were followed as previously. The final single-point electronic energy correction was performed using the DLPNO–CCSD(T)/Def2-QZVPP method as the DLPNO method has been shown previously to compare well with measurements for cluster binding energies.^96^

The temperature-dependent rate coefficients were calculated using the master equation solver for multienergy well reaction (MESMER) program.^97^ The formation of reactive complexes (RCs) was treated using the MesmerILT method with a pre-exponential factor of 2 × 10^–10^ cm^3^ molecule^–1^ s^–1^ and a modified Arrhenius parameter of 0.1. For the RC, an average energy transfer per collision with bath gas, *E*_down_, of 225 cm^–1^ was used, which is within the MESMER recommended range of 175–275 cm^–1^ for N_2_. The SimpleRRKM method was used for the formation of the sulfuric anhydride products, which were treated as “sink” during the simulations. The MESMER input and output files are provided as Supporting Files.

## 3 Results and Discussion

### 3.1 Formation and Detection of SO_3_ in Experiment

**Figure 1:**
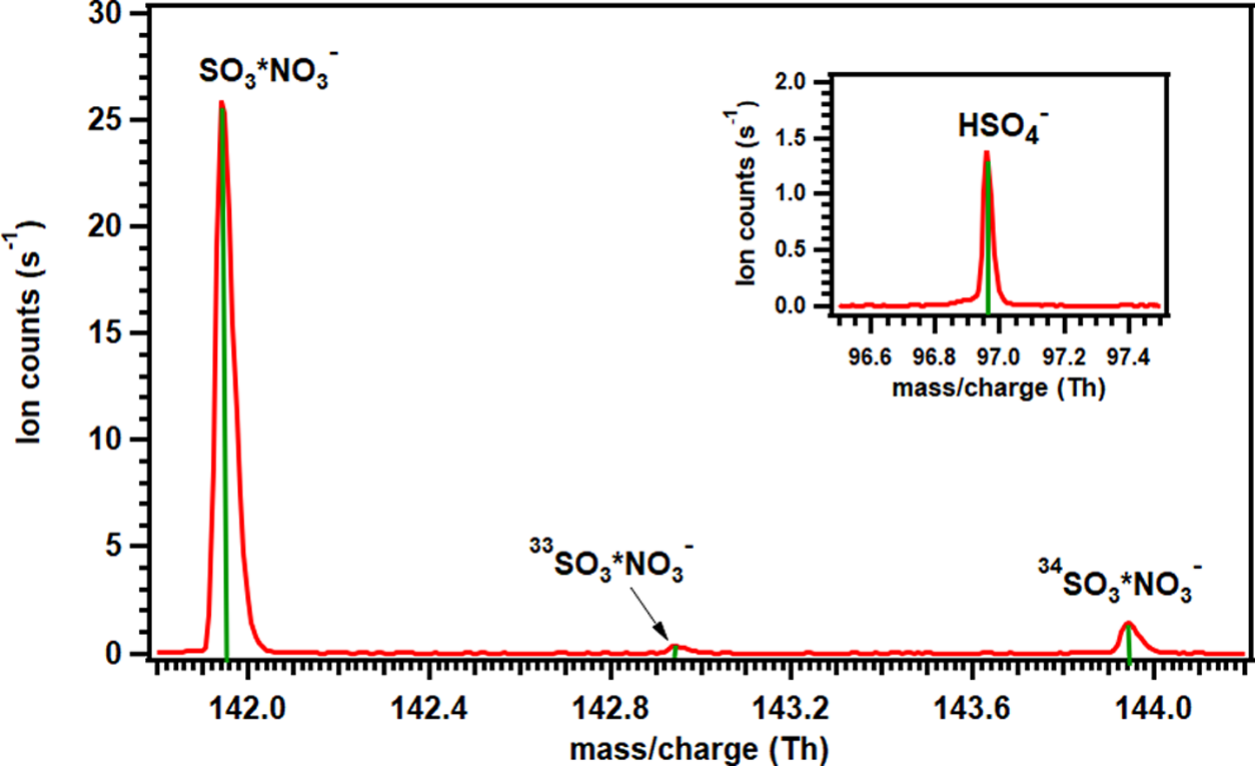
Mass spectra showing the formation of SO_3_ in the reaction system.

SO_3_ is not commercially available, and therefore it was produced in situ in the flow reactor by the reaction of SO_2_ with OH radical and sCI. OH radical and sCI (C_3_H_6_O_2_) were produced in situ by the reaction of TME with ozone, along with acetone as a side product (R1)^98,99^
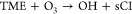
R1SO_2_ can react with produced OH and sCI to form SO_3_ in the presence of oxygen via reactions R2–R4^100−103^
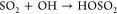
R2
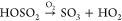
R3
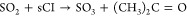
R4The formed SO_3_ was directly detected by NO_3_^–^-CIMS as SO_3_*NO_3_^–^ cluster with a nominal mass-to-charge ratio of 141.9452 Th (see Figure 1). Peaks corresponding to other sulfur (^33^S and ^34^S) isotopes,^104^ i.e., ^33^SO_3_*NO_3_^–^ and ^34^SO_3_*NO_3_^–^ with the nominal mass-to-charge ratio of 142.9446 and 143.9410 Th, respectively, were also measured.

We also observed trace levels of SA in our spectra with signals that were generally around 15 times lower than those of SO_3_*NO_3_^–^. The formation of SA may be due to the reaction of SO_3_ with water, which could have either formed chemically (from H-abstraction reactions of OH radicals with, e.g., precursor acids, TME, or acetone) or was present on the reactor surfaces or in the gas lines. The flows and concentrations in the chemical system were optimized to get a maximum signal of SO_3_ and the minimum possible of SA. The final concentrations of SO_2_, TME, and ozone were maintained at 2.73 × 10^15^, 2.89 × 10^11^, and 1.67 × 10^11^ molecules cm^–3^, respectively. The same concentrations of these three SO_3_ precursors were used for all of the experiments. To detect the produced SO_3_ with nitrate ionization, Yao et al.^73^ have calculated the electronic binding energy of SO_3_*NO_3_^–^ to be −44.4 kcal mol^–1^, which is substantially higher than that of HNO_3_*NO_3_^–^ (−29.2 kcal mol^–1^), leading to efficient detection by NO_3_^–^-CIMS.^105^ Therefore, nitrate ionization was used in this study to detect SO_3_ as well as the reaction products.

### 3.2 Ambient Field Measurement of SO_3_

**Figure 2:**
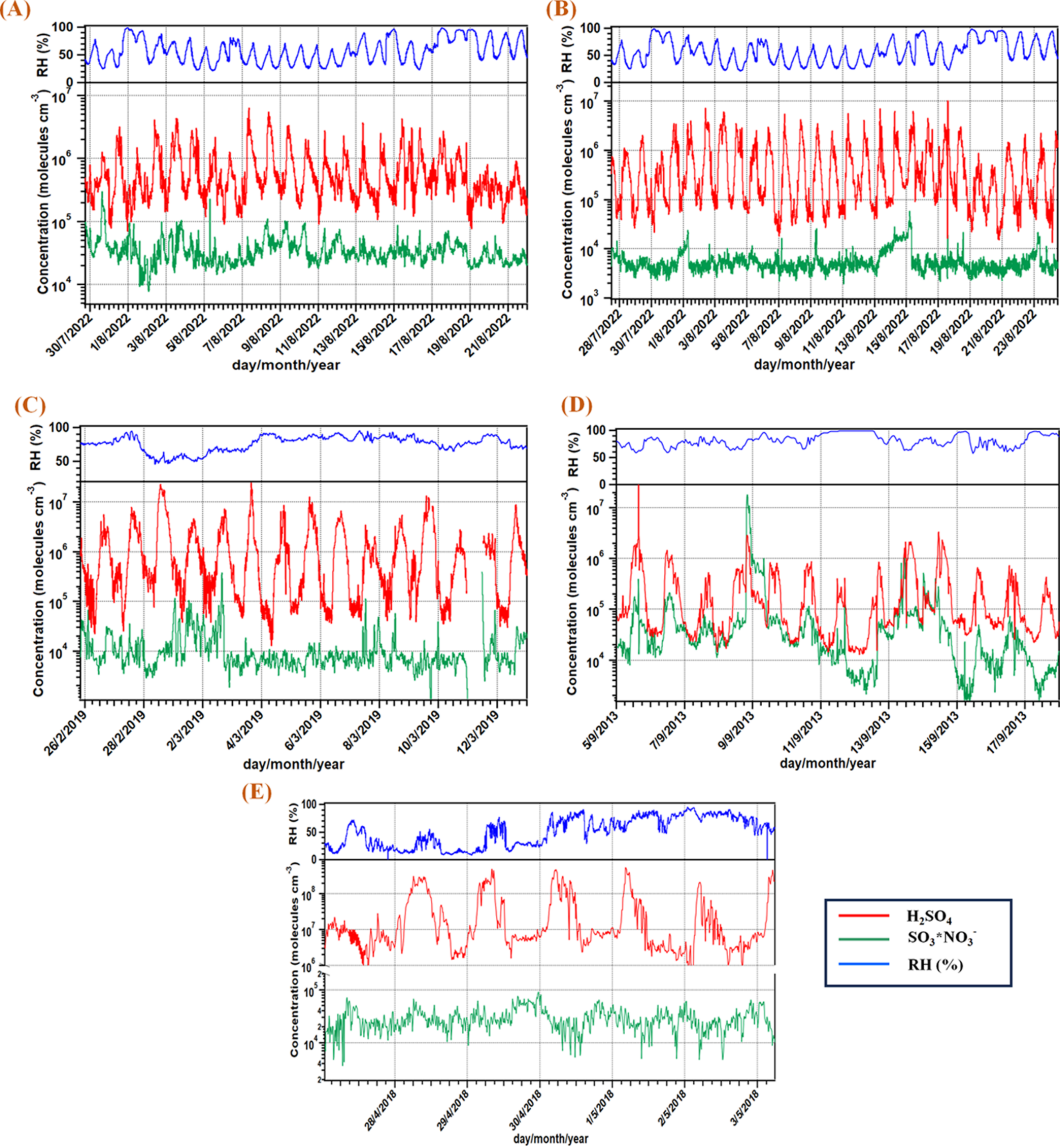
Time series plots showing the variation in the concentration of SO_3_ and H_2_SO_4_ at contrasting chemical environments: (A) urban roadside site measurement station in Leipzig, Germany; (B) urban background site measurement station located at TROPOS, Leipzig, Germany; (C) Spanish research station Juan Carlos I, Antarctic Peninsula; (D) Mace Head research station, Ireland; and (E) Maïdo Observatory, Réunion island. All of the signals are normalized to the reagent ion signals. High-resolution peak fits of the individual products (SO_3_*NO_3_^–^, HSO_4_^–^, and HNO_3_*HSO_4_^–^) are shown in the Supporting Information (Figures S14–S18). The concentration of H_2_SO_4_ in the Mace Head research station (D) is from the contribution of the HNO_3_*HSO_4_^–^ signal only due to an overlapping peak in the HSO_4_^–^ signal (see Section S1.5.4 in the Supporting Information), whereas for all other locations, both the signals of HSO_4_^–^ and HNO_3_*HSO_4_^–^ are taken into account.

Gas-phase SO_3_ was observed at various locations, spanning from urban roadside and background sites in Leipzig, Germany, the polar Spanish research station Juan Carlos I in Antarctica, the marine Mace Head research station at coastal Ireland, and also the Maïdo Observatory located on Réunion island during volcanic plume conditions. The descriptions of these research locations are comprehensively detailed in the Supporting Information (S1.5). Measurement of SO_3_ was performed by NO_3_^–^-CIMS, and the diurnal profiles of SO_3_ and SA concentrations during the select days at the different measurement sites are shown in Figure 2. The average measured concentration of SO_3_ mostly varied from 10^4^ to 10^5^ molecules cm^–3^ at all locations except Mace Head where on some days SO_3_ was as high as 10^7^ molecules cm^–3^ (Figure 2D). The time period for Maïdo Observatory illustrated in Figure 2E pertains specifically during the volcanic eruption when SO_2_ concentration at the measurement site was close to 100 ppb at its highest.^106^ The prevalence of SO_3_ in such diverse chemical environments suggests the hitherto unprecedented occurrence of its bimolecular reaction with other atmospherically relevant species, such as organic and inorganic acids.

### 3.3 Reaction of SO_3_ with Monocarboxylic Acids

**Figure 3:**
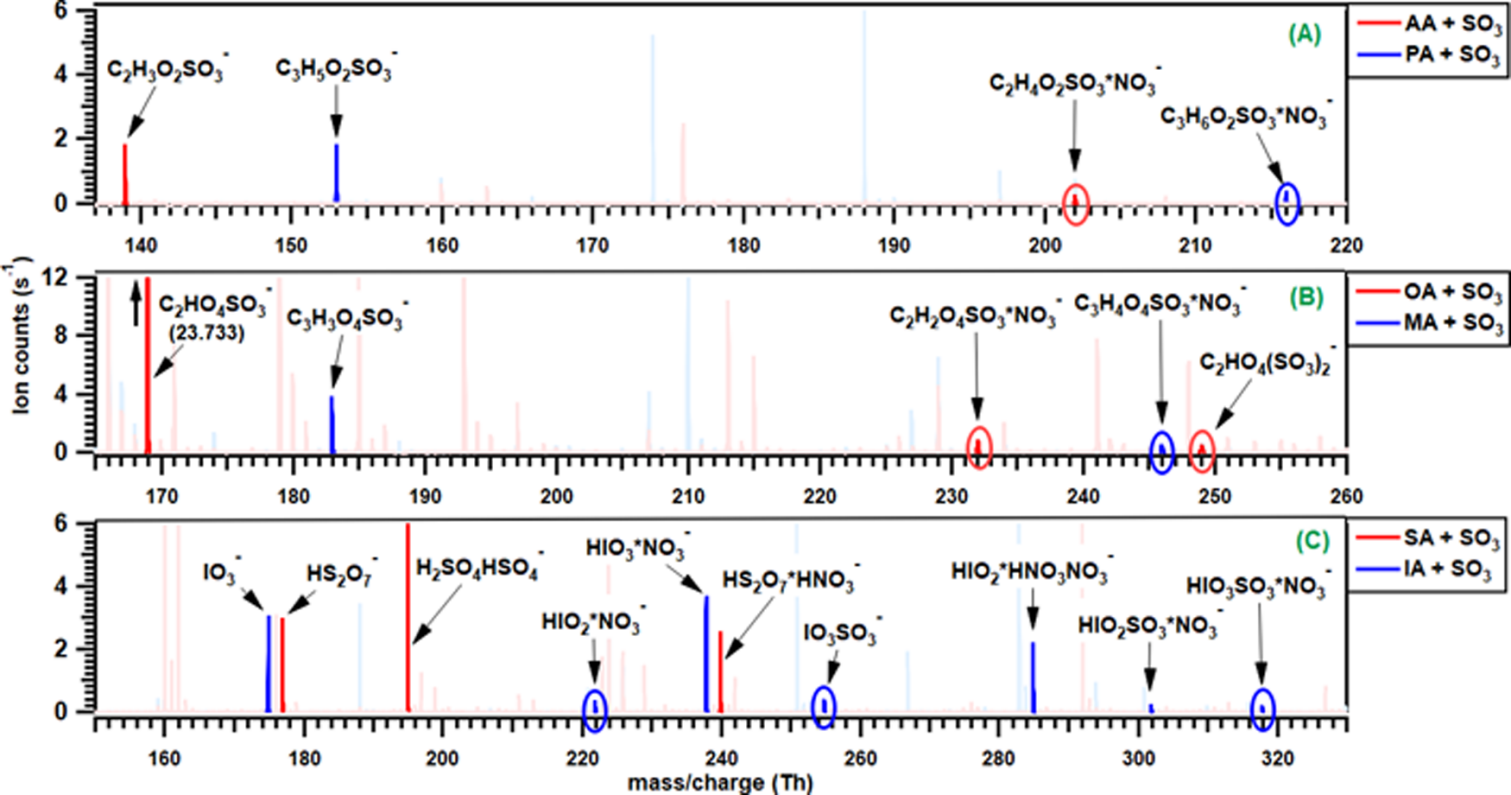
NO_3_^–^-CIMS mass spectra obtained in the reaction of SO_3_ with monocarboxylic acids (AA and PA) (panel A), dicarboxylic acids (OA and MA) (panel B), and inorganic acids (SA and IA) (panel C). The signals shown with a lower intense color in the background are the unidentified peaks. Note the different scales of the *y*-axes and the different mass range coverages. High-resolution peak fitting for all of the product peaks is given in the Supporting Information (Figure S13). Note that each of these mass spectra was obtained individually, with only one acid present at a time.

**Figure 4:**
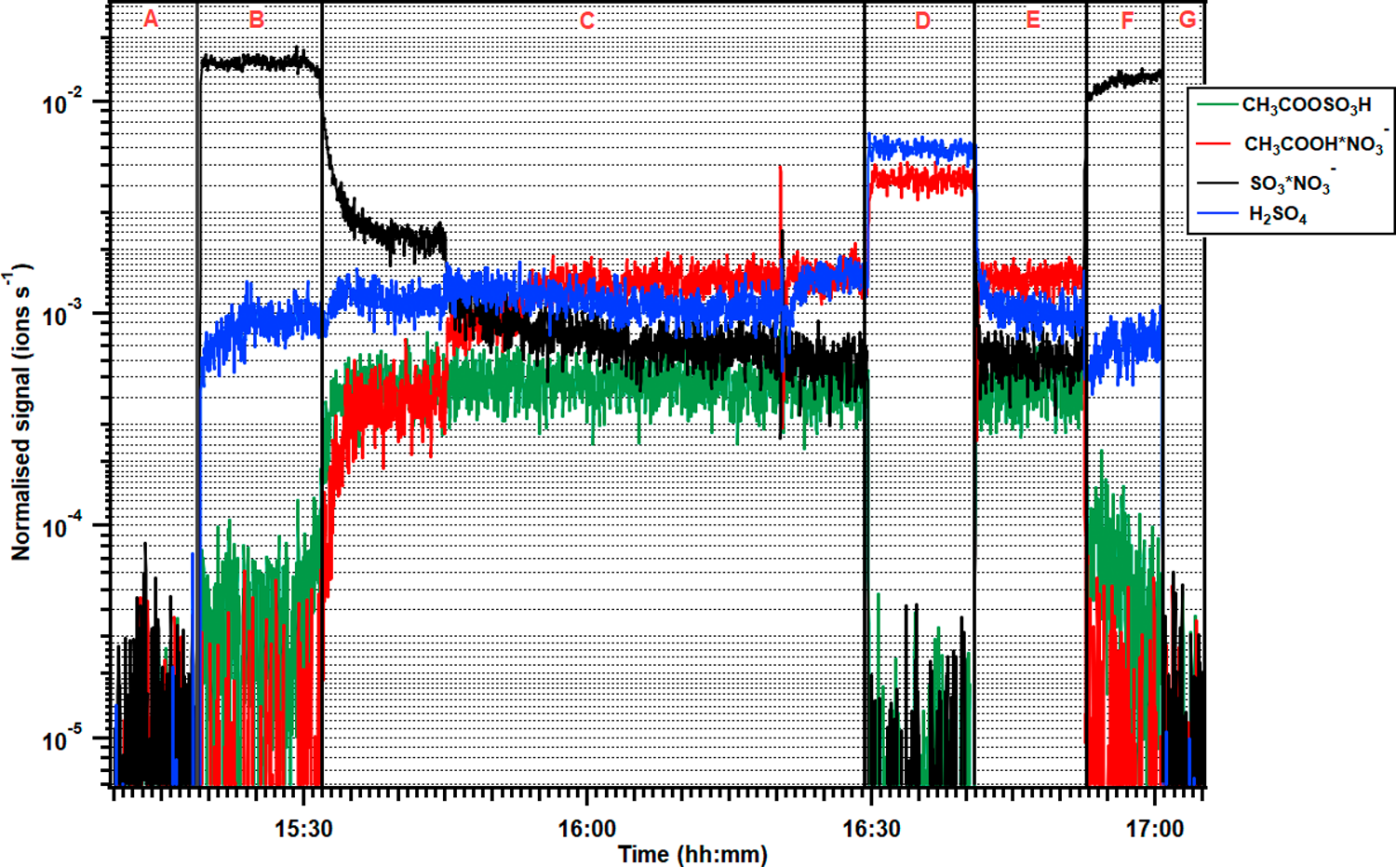
Normalized time series plot obtained during the reaction of acetic acid with SO_3_. (A) Background; (B) injection of SO_2_ and OH radical (formation of SO_3_); (C) injection of acetic acid; (D) injection of water in the reaction system; (E) stopped injection of water; (F) stopped injection of acetic acid; and (G) stopped ozone (no OH radical). The normalized signals of CH_3_COOSO_3_H and H_2_SO_4_ are represented as (*S*_CH_3_COOSO_3_^–^_ + *S*_CH_3_COOSO_3_H*NO_3_^–^_) and (*S*_HSO_4_^–^_ + *S*_HNO_3_*HSO_4_^–^_), respectively. The concentration profile for acetic acid does not reveal its actual concentration due to the poor sensitivity of the NO_3_^–^ reagent ion toward acetic acid.

The reaction of monocarboxylic acids, namely, acetic acid (AA) and propionic acid (PA) with SO_3_, produced acetic sulfuric anhydride (ASA) and propionic sulfuric anhydride (PSA), respectively (Figure 3A). The computed reaction rate coefficients and the energetics of the clustering pathways enabling the detection of these products by NO_3_^–^-CIMS is provided in Table 1. These products were formed by the cycloaddition reaction mechanism of SO_3_ to AA or PA, where the acidic hydrogen atom is transferred to one of the O atoms of the SO_3_ molecule accompanied by a simultaneous formation of O (from acid) and S bonds (details are given in Section 3.5). Along with the CSA*NO_3_^–^ clusters, deprotonated signals of ASA (CH_3_COOSO_3_^–^) and PSA (C_2_H_5_COOSO_3_^–^) were also observed in our spectra. The product signal intensities showed an increasing trend when the concentrations of the acids were ramped up. With the addition of water (∼10^16^ molecules cm^–3^) to the reaction system, the signal corresponding to ASA and PSA dropped. This can be attributed to the fact that upon the addition of sufficient water, all of the SO_3_ gets converted to SA and hence diminishes the product signal. This can be verified by the decrement of the SO_3_ signal intensity and a corresponding increase in the SA signal intensity (Figure 3). Upon the addition of water, the signal intensity corresponding to the parent AA (or PA) increases, which is due to less consumption in the reaction with SO_3_ in the system. These shreds of evidence confirm the generation of SO_3_ in our reaction system and show that ASA (or PSA) is formed solely due to the reaction of SO_3_ with AA (or PA). The normalized time series plot indicating the variation of the signal intensities of the products and reactants in the AA-SO_3_ reaction system is shown in Figure 4, and for all other reaction systems, it is shown in the Supporting Information (Figures S8–S12). The mass spectra showing the effect of water for all of the studied reaction systems are shown in Figures S2–S7 in the Supporting Information.

**Table 1:** Calculated Rate Coefficients and Fragmentation and Deprotonation Enthalpies of the Formed Products along with Their Exact Mass-to-Charge Ratios^a^

reaction system (acid + SO_3_ → X)	*k*^b^ (cm^3^ molecule^–1^ s^–1^)	Δ*H*_a_ (kcal mol^–1^) (X*NO_3_^–^ → X+ NO_3_^–^)	Δ*H*_b_ (kcal mol^–1^) (X*NO_3_^–^ → X^–^ + HNO_3_)	product species	exact mass-to-charge ratio (*m*/*z*; Th)^f^
	1.37 × 10^–10^	40.73	20.88	C_2_H_3_O_2_SO_3_^–^	138.9707
				C_2_H_3_O_2_SO_3_H^e^	201.9663
	1.60 × 10^–10^	40.28	20.79	C_3_H_5_O_2_SO_3_^–^	152.9863
				C_3_H_5_O_2_SO_3_H^e^	215.9820
	9.72 × 10^–12^^c^	50.82	17.62	C_2_HO_4_SO_3_^–^	168.9448
				C_2_HO_4_SO_3_H^e^	231.9405
	3.40 × 10^–11^^d^	59.04	12.95	C_2_HO_4_(SO_3_)_2_^–^	248.9017
	5.83 × 10^–12^^c^	49.87	16.56	C_3_H_3_O_4_SO_3_^–^	182.9605
	7.70 × 10^–12^^d^	51.38	12.87	C_3_H_3_O_4_SO_3_H^e^	245.956
	2.75 × 10^–11^	53.18	16.72	HS_2_O_7_^–^	176.9169
				H_2_S_2_O_7_^e^	239.9126
	5.43 × 10^–11^	51.10	21.59	IO_3_SO_3_^–^	254.8466
				IO_3_SO_3_H^e^	317.8422

a Δ*H*_a_ – fragmentation enthalpy. Δ*H*_b_ – deprotonation enthalpy.

b Rate coefficient at 1 atm. pressure and 298 K.

c Rate coefficient for the primary addition of SO_3_.

d Rate coefficient for the secondary addition of SO_3_.

e Peaks detected as clusters with NO_3_^–^.

f Mass-to-charge ratio given in Thomson unit.

### 3.4 Reaction of SO_3_ with Dicarboxylic Acids

A similar set of experiments were performed for the reaction of SO_3_ with oxalic (OA) and malonic acids (MA). In contrast to monocarboxylic acids, now there is a possibility of SO_3_ addition to both carboxylic groups of the same molecule, which leads to the formation of secondary products, namely, oxalic/malonic disulfuric anhydride (ODSA/MDSA) along with the primary products, oxalic/malonic monosulfuric anhydride (OMSA/MMSA). The concentrations of the acids were increased while maintaining the same concentration of SO_2_, TME, and ozone in the reaction system to verify the product signal, which shows the corresponding increase in their respective signal intensity. In the OA-SO_3_ system, the deprotonated signal was observed for both OMSA and ODSA, whereas the NO_3_^–^ cluster for ODSA was not observed. It is revealed from the spectra as well as from quantum chemical calculation (vide infra) that these CSAs are prone to deprotonation, and the signal will be more intense than its nitrate cluster. In the MA-SO_3_ reaction system, we did not observe any secondary products, i.e., MDSA in both deprotonated and cluster forms. Malonic acid is prone to undergo keto–enol tautomerism, forming a distinct structural isomer with only one −C(O)OH group (i.e., the enol form). The formation of an enol form of malonic acid can be self-catalyzed and can be stabilized by water molecules.^107−109^ Since the enolic form of malonic acid has only one acid functionality, the formation of MDSA could be susceptible to the formation of the enol form. It could be seen in the mass spectra of the OA-SO_3_ system (Figure 3B) that the signal intensity of deprotonated ODSA is nearly 25 times lower than deprotonated OMSA and in the case of the MA-SO_3_ system, the intensity of the primary product (MMSA) is itself low and therefore the secondary product, i.e., MDSA was not detected. This lower intensity of MMSA is attributed to the lower concentration of MA in the reaction system due to its lower vapor pressure and the lower rate coefficient of the reaction between MA and SO_3_, which is nearly half that of the OA-SO_3_ reaction system, as the detection characteristics should be similar for both MMSA and OMSA (Table 1). The mass spectra showing the product peaks resulting from the reaction of OA and MA with SO_3_ are shown in Figure 3B.

### 3.5 Reaction of SO_3_ with Inorganic Acids

We performed the experiments for the reaction of SO_3_ with inorganic acids, namely, sulfuric acid (SA) and iodic acid (IA). Disulfuric acid (DSA) was formed in the reaction of SA with SO_3_, and the obtained mass spectrum is shown in Figure 3C. SA was bubbled into the reaction system, and the formed DSA was observed as a deprotonated cluster with nitric acid (HNO_3_*HS_2_O_7_^–^) as well as in its deprotonated form (HS_2_O_7_^–^). DSA signal intensity shows an increasing trend with an increase in the concentration of SA.

IA was produced in situ in the reaction system by photolysis of iodine (I_2_) with subsequent reaction with ozone, which initially forms IO radical. The formed IO radical further oxidizes to higher oxides, which in the presence of OH or O_3_/H_2_O generates IA (HIO_3_).^59,110^ The detailed mechanism for the formation of iodic acid and iodous acid (HIO_2_, IoA) is given in the Supporting Information (S1.4). The formed iodic acid reacts with SO_3_ to form iodic sulfuric anhydride (ISA), and the obtained mass spectrum is shown in Figure 3C. As observed in all of the acid + SO_3_ systems inspected here, the ISA is also detected both in its deprotonated form (IO_3_SO_3_^–^) and as a cluster with NO_3_-(IO_3_SO_3_H*NO_3_^–^). Interestingly, we also observed a signal corresponding to iodous sulfuric anhydride (IO_2_SO_3_H, IoSA) clustered to NO_3_^–^ along with iodous acid (HIO_2_, IoS), the HIO_2_ being recently identified as an important contributor to iodine-related ambient particle formation events.^65,111^

To the best of our knowledge, this is the first experimental observation of gas-phase DSA, ISA, and IoSA under atmospheric pressure and room temperature. When the water was added to both the reaction systems (H_2_SO_4_–SO_3_ and HIO_3_–SO_3_), the signals corresponding to DSA and ISA plummeted, indicating that the formation of these products is solely due to the reaction of SO_3_ with SA and IA, respectively.

### 3.6 Kinetic Calculation

**Figure 5:**
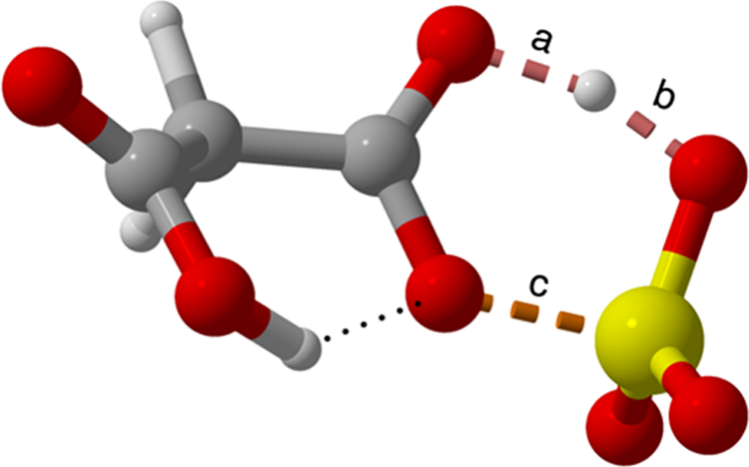
Transition-state geometry of malonic acid + SO_3_ with the relevant bonds labeled.

The addition of SO_3_ is an exergonic process for all studied systems, besides oxalic acid. In all cases, they first form a reaction complex (RC) before proceeding to form the product (P) via the transition state (TS). The TS involves the migration of the acidic H atom to one of the oxygen atoms of SO_3_ while simultaneously forming a bond between the carbonyl O atom of the acid and the sulfur atom. The TS structure for malonic acid is shown in Figure 5, with the bonds participating in the reaction labeled. Table 2 shows the corresponding bond lengths of the optimized TS structure for the studied acids and the energies of all of the reaction stationary points relative to the separated reactants.

**Table 2:** Bond Lengths of the Optimized TS Geometries and Energetics of All Stationary Points of the Studied Systems at the RHF-RCCSD(T)-F12a/VDZ-F12//ωB97X-D/aug-cc-pV(T+d)Z Level of Theory

	bond length (Å)			Δ*G* kcal/mol (relative to separated reactants)		
acid	*a*	*b*	*c*	RC	TS	*P*
acetic acid (AA)	1.20	1.20	1.76	–4.6	–2.5	–5.5
propionic acid (PA)	1.20	1.20	1.76	–3.9	–2.4	–6.2
oxalic acid (OA)	1.19	1.21	1.81	+2.9	+5.3	+0.4
malonic acid (MA)	1.21	1.19	1.80	+1.3	+5.1	–3.7
sulfuric acid (SA)	1.15	1.27	1.79	–0.2	+1.6	–6.0
iodic acid (IA)	1.14	1.28	1.79	–1.6	–0.9	–10.0

**Figure 6:**
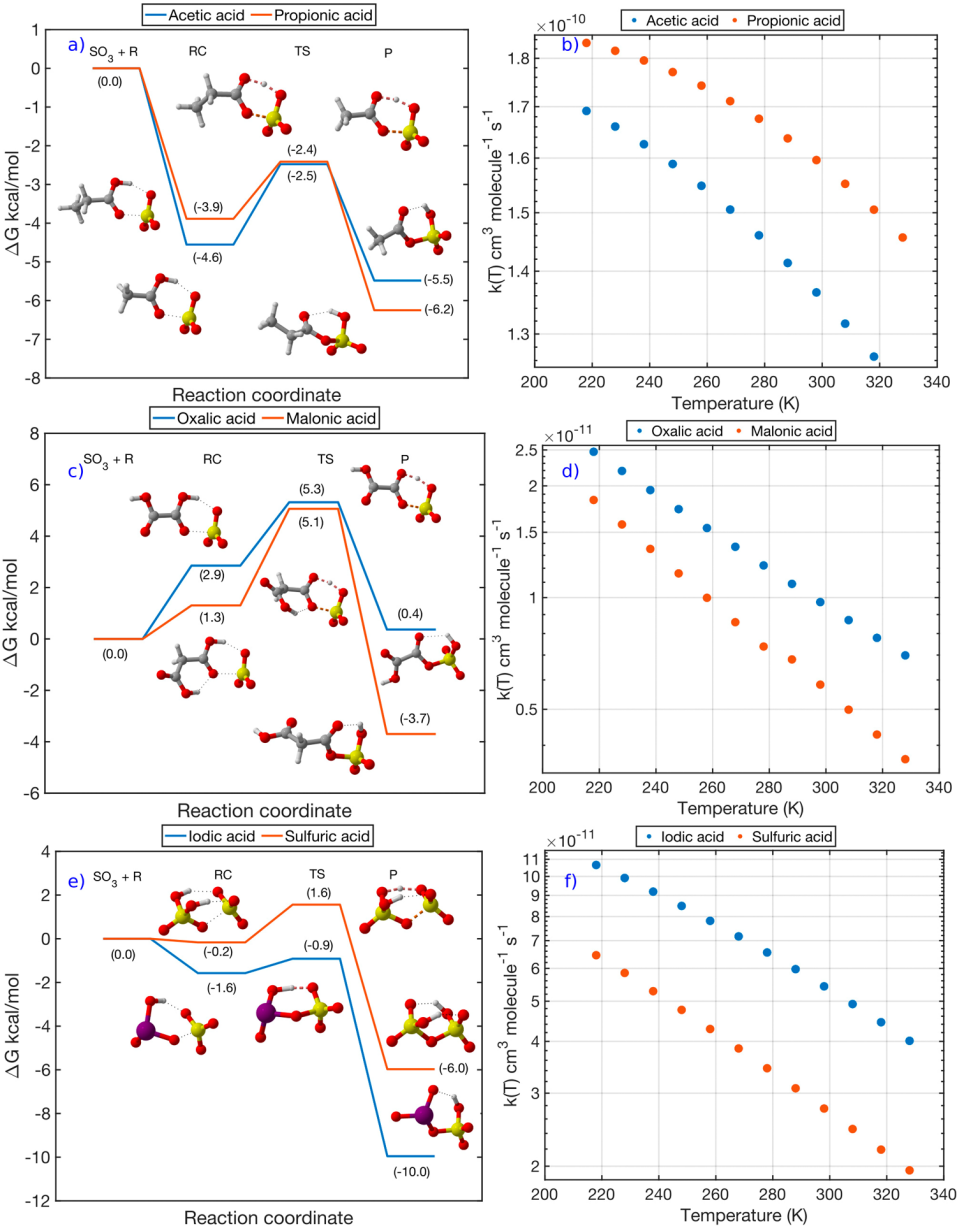
Potential energy surface and computationally calculated temperature-dependent rate coefficients for the bimolecular reaction of SO_3_ with the studied (a, b) carboxylic acids, (c, d) dicarboxylic acids, and (e, f) inorganic acids. The energies shown in the potential energy surfaces are calculated at the RHF-RCCSD(T)-F12a/VDZ-F12//ωB97X-D/aug-cc-pV(T+d)Z level of theory. Color coding for the atoms: sulfur, yellow; oxygen, red; hydrogen, white; and iodine, purple.

Interestingly, the studied dicarboxylic acids have significantly lower rate coefficients with SO_3_ relative to the other acids, especially the monocarboxylic acids. In contrast to the monocarboxylic acids, the free dicarboxylic acid molecules have two intramolecular H-bonds instead of one. Significantly, one of the H-bonds is lost when the corresponding RC and TS structures for the studied dicarboxylic acids. This leads to higher RC and TS energies for the dicarboxylic acids compared to the other studied acids (see Figure 5), which also holds for the second SO_3_ addition reaction (Table S3 in the Supporting Information). The opposite is true for the monocarboxylic acids, which have more stable RCs and lower TS energies relative to those of the dicarboxylic acids. This translates to extremely fast reactions with SO_3_, with rate coefficients that are close to the collision limit. The rate coefficients of the studied inorganic acids are also very fast and lie between those of the monocarboxylic and dicarboxylic acids. Both H atoms of sulfuric acid form H-bonds with SO_3_ in the RC geometry, with only one H atom transferred in the TS similar to that of the other acids. Following a similar reaction, the ISA product of iodic acid has the lowest energy relative to the separated reactants of all of the studied acids. Note that all of the studied acids react with SO_3_ sufficiently fast to be relevant for atmospheric chemistry (Figure 6).

### 3.7 Detection of CSA, DSA, and ISA by NO_3_^–^-CIMS

The calculated binding enthalpies indicate that the sulfuric anhydride products form strong clusters with NO_3_^–^ ions that are stable against fragmentation back into the reactants but can nevertheless deprotonate. This is in agreement with the observed deprotonated and cluster peaks in the NO_3_^–^-CIMS spectra, with the former dominating for all of the studied acids. The computed enthalpies are listed in Table 1.

### 3.8 Atmospheric Implications

**Figure 7:**
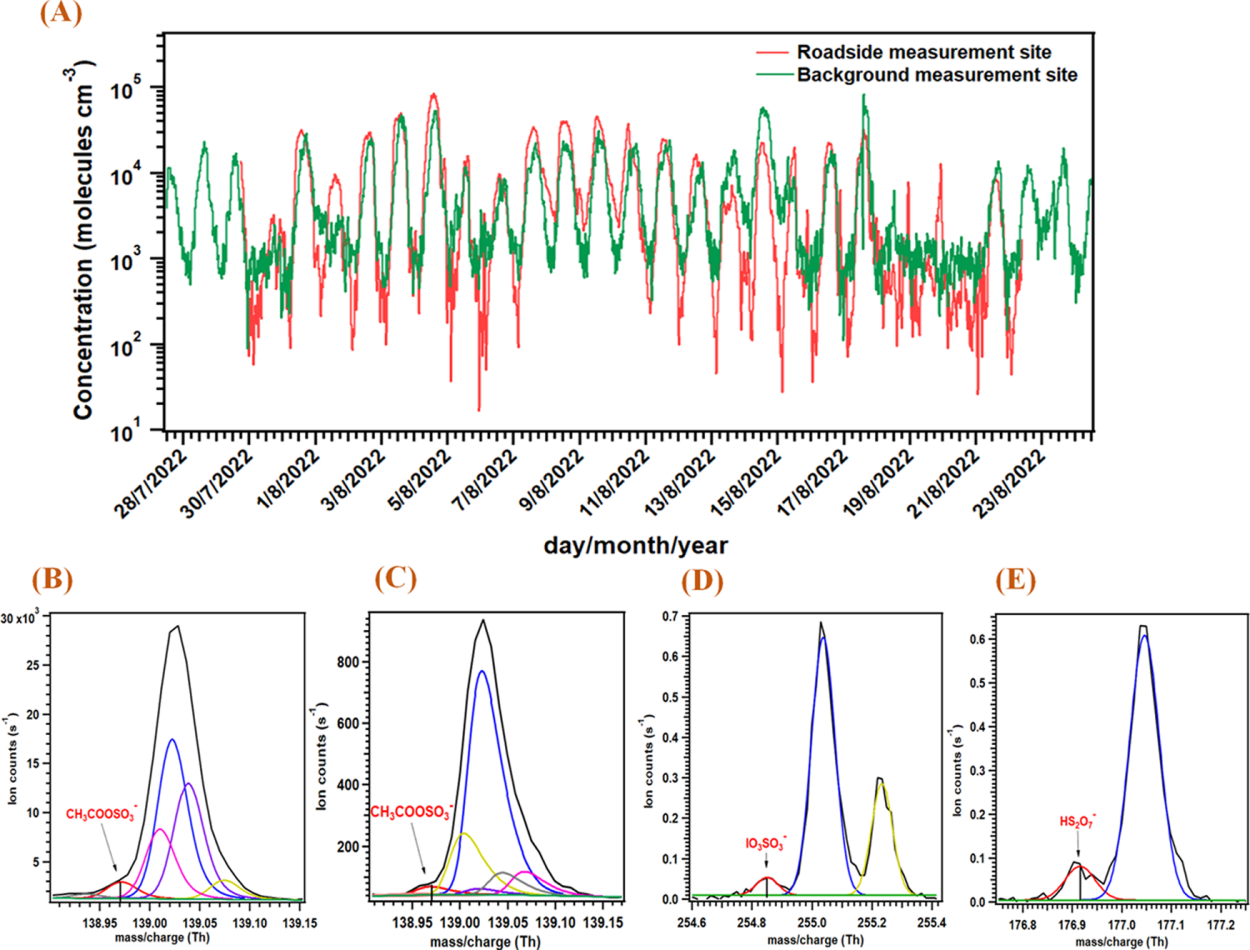
Time series of acetic sulfuric anhydride (deprotonated form; CH_3_COOSO_3_^–^) measured at urban roadside and background sites in Leipzig, Germany (A); high-resolution peak fit of acetic sulfuric anhydride (deprotonated form; CH_3_COOSO_3_^–^) measured at the urban roadside (B) and background (C) sites in Leipzig, Germany; deprotonated iodic sulfuric anhydride (ISA; IO_3_SO_3_^–^) measured at Mace Head research station, Ireland (C); and deprotonated disulfuric acid (DSA; HS_2_O_7_^–^) measured at Mace Head research station, Ireland (D). Black and red color traces in the spectra (B–E) represent the raw spectra and high-resolution fitted peak for the ion of interest, respectively.

Previous studies have indicated that the reaction of SO_3_ with water dimer to form sulfuric acid is the dominant loss pathway of gaseous SO_3_ in the troposphere.^112−116^ The concentration of water can range from 10^12^ to 10^17^ molecules cm^–3^ under tropospheric conditions,^117^ and the experimental rate coefficient for the conversion of SO_3_ to SA via its reaction with water in the temperature range of 250–360 K is (2.26 ± 0.85) × 10^–43^*T* exp ((6544 ± 106)/*T*) [H_2_O]^2^ s^–1^.^114^ This results in an apparent SO_3_ tropospheric lifetime of only around 10^–5^ s with respect to its reaction with water dimer. We compared this to the lifetime of SO_3_ with respect to its reaction with the studied acids using the temperature-dependent rate coefficients computed in this study. Table S4 (in the Supporting Information) compares the lifetime of SO_3_ against its reaction with the water dimer and the studied acid molecules at RH = 20 and 40%. The calculated lifetime values indicate that the dominant loss of SO_3_ is its reaction with the water dimer, with reaction with propionic acid accounting for a maximum 6% of SO_3_ loss at *T* = 275 K. The loss of SO_3_ to the remaining acids ranges from 5% to completely negligible. Despite this, we should highlight that gaseous SO_3_ was nevertheless measured at concentrations above 10^6^ molecules cm^–3^ during winter in Beijing, China.^73^ Moreover, as shown in Figure 2, SO_3_ is also detected in significant concentrations in high-altitude marine environments, volcanic plumes, urban measurement sites, and polar regions. Interestingly, the signal related to CSA, particularly, acetic sulfuric anhydride (CH_3_COOSO_3_^–^), was detected in the urban region at both roadside and background measurement sites in Leipzig, Germany (Figure 7A–C), and ISA and DSA were detected in the marine environment at Mace Head research station, Ireland (Figure 7D,E). To the best of our knowledge, this is the first such report of the gas-phase detection of CSA, ISA, and DSA in urban and marine air samples.

The peak corresponding to SO_3_ that was persistently overlooked is seen in many of our field data as shown in Figure 2. The tool of choice for many such field measurements worldwide, NO_3_^–^-CIMS, just happens to detect SO_3_ extremely well, but due to the accepted paucity of gas-phase SO_3_ under ambient conditions, the peak remained unidentified (Yao et al.,^73^ a notable exception). This ubiquity of SO_3_ in the ambient environment indicates that there are large gaps in our understanding of its sources and sinks and makes its reactions besides those with the water dimer of paramount importance. Our kinetic calculations indicate that the reaction with SO_3_ is extremely rapid for all of the studied acids and the hydrolysis of the formed CSA is likely a route of incorporation of the (small) carboxylic acids into aerosol droplets, which is consistent with the previously published speculation.^118^ The CSAs have strong nucleation potential and are potential participants in new particle formation in the atmosphere.^84−89^ DSA can accelerate the NPF process from SA-NH_3_-based clusters, and at the air–water interface, the deprotonated DSA can accelerate particle growth by attracting gas-phase species to the liquid surface.^90^ The detection of gas-phase SO_3_ in the highly humid and iodine-rich air over the Mace Head Atmospheric Research Station, Ireland, is particularly significant as this allows its interplay with the major NPF precursor, iodic acid, confirming the relevance of this reaction class in marine environments and coastal mega-cities.

## 4 Conclusions

This work exemplifies the gas-phase reaction between sulfur trioxide, SO_3_, and common atmospheric acids including mono- and dicarboxylic acids, sulfuric acid, and iodic acid to form carboxylic sulfuric anhydrides, disulfuric acid, and iodic sulfuric anhydride, respectively, under tropospheric relevant conditions of temperature and pressure. The experiments showing the formation of sulfuric anhydrides from various carboxylic acids were reported previously. However, none of the previously reported experiments measured the products at atmospheric pressure and room temperature. The theoretical rate coefficient calculations show that these reactions are competitive to the reaction of SO_3_ with water dimer and should be considered as loss processes of SO_3_ in the atmosphere. Our calculations show that NO_3_^–^ ionization coupled to CIMS is a sensitive method to detect the sulfuric anhydrides, dominantly as deprotonated ions, which was also verified by our experimental investigation. The present findings also show that these acid-derived organosulfur compounds can be formed via gas-phase reactions followed by their incorporation into atmospheric nanoparticles. This has a direct relevance to understanding the atmospheric aerosol sulfur content, which is usually considered to be formed via multiphase reactions. These reactions advance our understanding of the organic/inorganic acid-sulfur oxides interactive chemistry in the atmosphere, and the inclusion of these reactions into atmospheric chemistry models appears critical for understanding their implication to aerosol formation, especially in highly polluted regions.

## Supporting Information Available

Details about the experimental setup and the chemical system; mass spectra of all of the studied reaction systems in the presence and absence of water; site and instrumentation descriptions of all of the field measurement sites; time series profiles of all of the studied reaction systems; high-resolution peak fitting of the product signals of all of the studied reactions; high-resolution peak fitting of all of the reported ions in the ambient measurement; time series profile of ISA and DSA measured at Mace Head research station, Ireland; the energy of the stationary points involved in the secondary SO_3_ addition to dicarboxylic acids; and atmospheric lifetimes (s) of SO_3_ with respect to its bimolecular reaction with water dimer and studied acids in the temperature range of 275–320 K (PDF)
