# Correction to “Direct Measurements of Covalently Bonded Sulfuric Anhydrides from Gas-Phase Reactions of SO_3_ with Acids under Ambient Conditions”

**DOI:** 10.1021/jacs.4c16560

**Published:** 2024-12-23

**Authors:** Avinash Kumar, Siddharth Iyer, Shawon Barua, James Brean, Emin Besic, Prasenjit Seal, Manuel Dall’Osto, David C. S. Beddows, Nina Sarnela, Tuija Jokinen, Mikko Sipilä, Roy M. Harrison, Matti Rissanen

The following note about data availability was omitted from the original publication:

## Data Availability

The output files for all the stationary points involved in the quantum chemical calculations are available online (10.5281/zenodo.10417876).

