# Supplementary material for: Direct Measurements of Covalently Bonded Sulfuric Anhydrides from Gas-Phase Reactions of SO3 with Acids under Ambient Conditions
Source: J Am Chem Soc. 2024 May 21;146(22):15562–75. doi: 10.1021/jacs.4c04531 (PMC11157540; doi:10.1021/jacs.4c04531)
Supplement: Supplementary file 1 — ja4c04531_si_001.pdf [file ja4c04531_si_001.pdf]

SUPPORTING INFORMATION

for

Direct measurements of covalently bonded sulfuric anhydrides from gas phase reactions of SO_3 with acids under ambient conditions

Avinash Kumar[1†*], Siddharth Iyer[1†*], Shawon Barua[1], James Brean[2], Emin Besic[1], Prasenjit Seal[1], Manuel Dall'Osto[3], David C. S. Beddows[4], Nina Sarnela[5], Tuija Jokinen[5,6], Mikko Sipilä[5], Roy M. Harrison[2] and Matti Rissanen[1,7]*

[1]Aerosol Physics Laboratory, Physics Unit, Faculty of Engineering and Natural Sciences, Tampere University, 33720 Tampere, Finland.

[2]School of Geography, Earth & Environmental Sciences University of Birmingham, Birmingham B15 2TT, United Kingdom.

[3]Institute of Marine Science, Consejo Superior de Investigaciones Científicas (CSIC), Barcelona 08003, Spain.

[4]National Centre for Atmospheric Science, School of Geography, Earth and Environmental Sciences, University of Birmingham, Edgbaston, Birmingham B15 2TT, UK.

[5]Institute for Atmospheric and Earth System Research (INAR)/Physics, Faculty of Science, University of Helsinki, P.O. Box 64, Helsinki, 00014, Finland.

[6]Climate & Atmosphere Research Centre (CARE-C), The Cyprus Institute, P.O. Box 27456, Nicosia, 1645, Cyprus.

[7]Department of Chemistry, University of Helsinki, PO Box 55, 00014, Helsinki, Finland.

*Corresponding author email: avinashkumar@tuni.fi, siddharth.iyer@tuni.fi and matti.rissanen@tuni.fi

† A. K. and S.I. contributed equally to this paper.

Contents:

S1. Experimental details: set up, chemicals and addition of water.	S4-S6
Figure S1: Schematic diagram of the experimental setup.	S4
Table S1: Concentration of the reactants used in the experiments	S5
S1.4. Production of iodic acid.	S6
S1.5. Field measurement site and instrumentation description	S7
Table S2: NO_3^--CIMS inlet flow (l min^{-1}) and calibration coefficient ($C_{H_2SO_4}$; molecules cm^{-3}) at different measurement locations.	S9
Figure S2: Mass spectra for the reaction of AA with SO_3 in the presence and absence of water.	S10
Figure S3: Mass spectra for the reaction of PA with SO_3 in the presence and absence of water.	S10
Figure S4: Mass spectra for the reaction of OA with SO_3 in the presence and absence of water.	S11
Figure S5: Mass spectra for the reaction of MA with SO_3 in the presence and absence of water.	S11
Figure S6: Mass spectra for the reaction of SA with SO_3 in the presence and absence of water.	S12
Figure S7: Mass spectra for the reaction of IA with SO_3 in the presence and absence of water.	S12
Figure S8: Normalized time series plot obtained during the reaction of PA with SO_3.	S13
Figure S9: Normalized time series plot obtained during the reaction of OA with SO_3.	S14
Figure S10: Normalized time series plot obtained during the reaction of MA with SO_3.	S15
Figure S11: Normalized time series plot obtained during the reaction of SA with SO_3.	S16
Figure S12: Normalized time series plot obtained during the reaction of IA with SO_3.	S17
Figure S13: High-resolution peak fitting of the product signals formed in all the studied reactions.	S18
Table S3: Second SO_3 addition to di-carboxylic acids. Energies of the stationary points are calculated at the ROHF-ROCCSD(T)-F12a/VDZ-F12//ωB97X-D/aug-cc-PV(T+d)Z level of theory.	S19
Figure S14. High-resolution peak fitting of A) SO_3*NO_3^- and B) HSO_4^- and C) HNO_3*HSO_4^- measured at the urban roadside measurement site in Leipzig, Germany. The black and red color trace represents the raw spectrum and fitted peak of the ion of interest respectively.	S19
Figure S15. High-resolution peak fitting of A) SO_3*NO_3^- and B) HSO_4^- and C) HNO_3*HSO_4^- measured at the urban background measurement site in TROPOS, Leipzig, Germany. The black and red color trace represents the raw spectrum and fitted peak of the ion of interest respectively.	S20

Figure S16. High-resolution peak fitting of A) $SO_3*NO_3^-$ and B) HSO_4^- and C) $HNO_3*HSO_4^-$ measured at the research station Juan Carlos I, Antarctic Peninsula. The black and red color trace represents the raw spectrum and fitted peak of the ion of interest respectively.	S20
Figure S17. High-resolution peak fitting of A) $SO_3*NO_3^-$, B) HSO_4^- and C) $HNO_3*HSO_4^-$ measured at the Mace Head research station, Ireland. The black and red color trace represents the raw spectrum and fitted peak of the ion of interest respectively.	S21
Figure S18. High-resolution peak fitting of A) $SO_3*NO_3^-$, B) HSO_4^- and C) $HNO_3*HSO_4^-$ measured at Maïdo Observatory, Réunion island. The black and red color trace represents the raw spectrum and fitted peak of the ion of interest respectively.	S21
Figure S19. Concentration time series plot for disulfuric acid ($HS_2O_7^-$) and iodic sulfuric anhydride ($IO_3SO_3^-$) measured at the Mace Head research station, Ireland.	S22
Table S4. Atmospheric lifetimes (s) of SO_3 with respect to its bimolecular reaction with water dimer and studied acids in the temperature range of 275 – 320K.	S22
References	S23

S1. Experiments:

S1.1. Experiment set up.

A chemical ionization atmospheric pressure interface time-of-flight mass spectrometer (CI-APi-TOF) was used to measure the product signals (Tofwerk Aerodyne Research Inc.) using an Eisele-type CI inlet.[1,2] HNO_3 vapors were introduced into the inlet by flowing a UHP N_2 gas through a concentrated HNO_3 which further ionizes to NO_3^- by exposure to soft X-rays (< 9.5keV, Hamamatsu L9490). The sheath air flow inside the inlet which contains unionized HNO_3 and NO_3^-, mixes with the sample gas flow and thus induces the ionization. The neutral HNO_3 collides with the NO_3^- to form $HNO_3{*}NO_3^-$, which charges the species in the sample gas flow. The charging of the species (X) or the formation of the adduct between the X and NO_3^- happens only if the bond formation enthalpy between X and NO_3^- is greater than the bond formation enthalpy of HNO_3 and NO_3^-.[3] If the species is a strong acid, the proton transfer mechanism dominates and will be detected as a deprotonated species (transferring H^+ to NO_3^-). The borosilicate glass flow tube (100 cm length and 4.7 cm i.d.) was connected to the CI-APi-TOF via the Eisele inlet as shown in Figure S1. The same experimental condition of pressure (1 atm) and temperature (T = 295 ± 2 K) was maintained throughout the experiments.

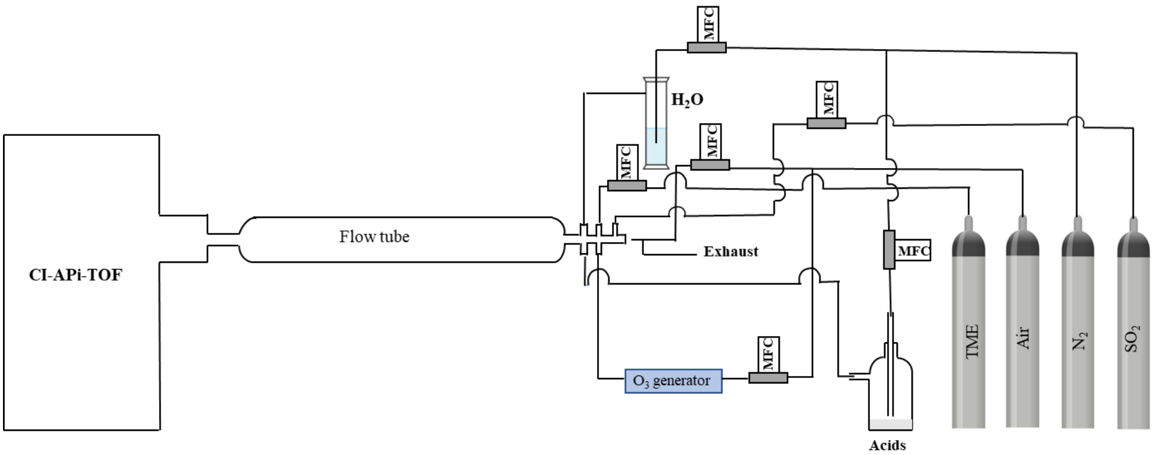

Figure S1. Schematic diagram of the experimental setup.

The concentrations of SO_2, TME and acids were calculated using the flow rates and the vapor pressures and the values are presented in Table S1. The concentration of produced O_3 was measured by the ozone monitor. The same experimental condition of pressure (1 atm) and temperature (T = 295 ± 2 K) was maintained throughout the experiments. PTFE tubings (6 mm o.d.) were used for carrying the flows of all the reagent vapors and gases to the flow reactor via a series of cross Swagelok fittings connected to the tail of the reactor whose internal diameter was contracted to ~7 mm, located at the distance of 5 cm upstream of the flow reactor.

The concentrations of all the reactants which were used during the experiments are given in Table S1.

Table S1. Concentration of the reactants used in the experiments.

**Reagent**	**Concentration (molecule cm^{-3})**
O_3	1.67×10^{11}
SO_2	2.73×10^{15}
TME	2.89×10^{11}
Acetic acid	$(0.72 - 1.79) \times 10^{14}$
Propanoic acid	$(1.47 – 7.34) \times 10^{13}$
Oxalic acid	$(0.45 - 1.35) \times 10^{11}$
Malonic acid	$(0.24 - 1.21) \times 10^{10}$
Sulfuric acid	$(0.65 – 2.89) \times 10^{12}$
Iodine crystal	$(0.77 – 5.44) \times 10^{12}$

All data processing, including averaging, mass axis calibration, and peak integration, was done using the tofTools software package for MATLAB. The signal intensity of all the mentioned species (X) was normalized using the following expression:

$$S = \frac{[X^{*}NO_3^-]}{[NO_3^-]+[HNO_3{}^{*}NO_3^-]+[(HNO_3)_2{}^{*}NO_3^-]} \quad \text{(I)}$$

S1.2. Chemicals

The SO_2 gas bottle of concentration 100 ppm in N_2 was obtained from Advanced Speciality gases whereas the UHP nitrogen gas (5.0 grade) was obtained from Woikoski Oy. A clean and dry zero air was obtained from the zero-air generator (AADCO Instruments, 737 Series) which was fed with clean in-house synthetic air. The generated zero air was further passed through a mist separator (SMC AMG 150C-F01C) and air membrane dryer (SMC IDG100LA-F04B-P) for further dehumidification connected upstream to airflow before the experimental setup. The O_3 was produced by the ozone generator (AnalytikaJena UVP) which is equipped with a Hg pen ray lamp (λ=184.9 nm). Pure water (HPLC grade) and concentrated HNO_3 (70%) were obtained from Sigma Aldrich which were bubbled by N_2 to the flow reactor and the inlet respectively. The acids - acetic (glacial, ACS reagent, purity ≥ 99.7%), propanoic (ACS

reagent, purity ≥ 99.5%), oxalic (anhydrous solid, purity 98%), malonic (anhydrous solid, purity 99%) and sulfuric acid (ACS reagent, 95.0–98.0%) - were commercially obtained from Sigma Aldrich. Iodine crystals (solid crystals, < 99.8% purity) were also obtained from the Sigma Aldrich. All the chemicals were used without further purification. All the flows were measured by calibrated mass flow controllers (Alicat Scientific).

S1.3. Addition of water

In our experiments, there was a formation of H_2SO_4 along with SO_3 which could have formed by the reaction of water with SO_3, even though the reactions were performed in dry conditions. To confirm the non-participation of H_2SO_4 in the formation of products (i.e., sulfuric anhydrides), water was added to the reaction system. Upon addition of water, all the SO_3 gets converted into H_2SO_4 (explained in the main manuscript), and the product signal drops. The mass spectra showing the absence of product peaks on the addition of water to the reaction system of SO_3 with AA, PA, OA and MA are shown in Figures S2-S7 respectively.

S1.4. Production of iodic acid (IA)

$$I_2 \longrightarrow 2\,I \qquad \text{(R5)}$$

$$I + O_3 \longrightarrow IO + O_2 \qquad \text{(R6)}$$

$$IO + IO \longrightarrow OIO + I \qquad \text{(R7)}$$

$$OIO + OH \longrightarrow HIO_3 \qquad \text{(R8)}$$

$$OIO + HO_2 \longrightarrow HIO_2 + O_2 \qquad \text{(R9)}$$

$$IO + IO \longrightarrow I_2O_2 \qquad \text{(R10)}$$

$$OIO + I \longrightarrow I_2O_2 \qquad \text{(R11)}$$

$$I_2O_2 + O_3 \longrightarrow IOIO_4 \qquad \text{(R12)}$$

$$IOIO_4 + H_2O \longrightarrow HIO_3 + HOI + O_2 \qquad \text{(R13)}$$

Iodine vapor was passed into the flow tube and photolyzed by using visible light to produce iodine radicals via R5. The iodine radical reacts with O_3 to form IO radical (via R6). IO radical forms OIO along with iodine radicals upon reacting with another IO (R7). The formed OIO reacts with OH radicals (produced in-situ by the reaction of TME and O_3) to form iodic acid (IA, HIO_3) via R8.[4] I_2O_2 formed by either self-combination of IO radicals or the reaction of OIO with iodine radicals (R10 and R11), reacts with O_3 to form $IOIO_4$ (R12). $IOIO_4$ in the presence of water can also be a source of IA along with HOI (R13).[5] HIO_2 can be formed by the reaction of OIO with HO_2 (R9).[5] HIO_2 shows a higher binding efficiency with $HNO_3NO_3^-$ than NO_3^-.[6]

S1.5 Measurement site and instrumentation description

S1.5.1. Urban Background, Leipzig, Germany

Measurements were conducted at an atmospheric research station operated by the Leibniz Institute for Tropospheric Research (TROPOS) within the Leipzig Science Park (51°21'09" N, 12°26'04" E), in the urban background setting. Measurements were taken from a south-facing window on the fourth floor of a research building, 14 m above ground level, ensuring a distance of more than 100 m from highly trafficked roads surrounding the site. The Leipzig Science Park is characterized by its diverse surroundings, including transport infrastructure (road, rail, tramways), commercial entities (restaurants, hotels, a petrol station, etc.), residential areas, on-street parking, and greenspaces, representing a typical urban environment with elevated condensation sink and black carbon concentrations.

The elemental composition of neutral molecules and clusters was analyzed with a CI-APi-TOF (chemical ionization-atmospheric pressure interface-time of flight) mass spectrometer (Tofwerk A.G) that was equipped with Eisele-type[8] nitrate CI inlet. The CI-APi-TOF was run in the negative ion mode and the signals detected are either negatively charged analyte ions (HSO_4^-) or analyte ion clustered with the charger ion (e.g. SO_3*NO_3^-). NO_3^- was produced by ionizing HNO_3 containing sheath gas with a soft X-ray source (Hamamatsu). Reagent ions were then introduced into the sample flow that travels in the center of the inlet with a flow rate of ~8 liters min^{-1}. The ion−molecule/cluster interaction time was ~200 ms. The reported signals were normalized with the sum of the charger ions and for concentration multiplied with calibration factor of (C_{H2SO4}) (Equation II). The calibration of the CI-APi-TOF was conducted with sulfuric acid that has been found to be ionized at the collision frequency with the reagent ion, NO_3^- using the updated methodology of Mettke et al.[7]

$$[X] = \frac{[X^*NO_3^-]}{[NO_3^-]+[HNO_3{}^*NO_3^-]+[(HNO_3)_2{}^*NO_3^-]} \times C_{H_2SO_4} \qquad \text{(II)}$$

The ion signal for SO_3*NO_3^- were converted to the concentration by using the same $C_{H_2SO_4}$ value due to similar sensitivities towards NO_3^- as that of H_2SO_4 as predicted by computed binding enthalpies.

The mass spectrometer used at other measurement sites was CI-APi-TOF with NO_3^- ionization. Hereafter, only the difference in the instruments parameter or method of measurement are highlighted, and the inlet flow and calibration coefficient are given in Table S2.

S1.5.2. Roadside, Leipzig, Germany

Measurements were conducted at a permanent observation site on Eisenbahnstraße, a major thoroughfare in the eastern part of the city (51°20'44” N, 12°24'23” E, 2 km from TROPOS), in the roadside environment. Measurements were taken from an apartment window 6 m above ground level on the northern side of the street, ensuring an observation point well integrated into the urban fabric. Eisenbahnstraße, with a width of approximately 20 m and flanked by multi-storey period buildings, presents an aspect ratio of 0.90 and accommodates about 12,000 vehicles per working day. The immediate vicinity of the observation site encompasses a two-lane roadway (one lane in each direction), an integrated tramline, on-street parking, bicycle lanes on both sides, pedestrian footpaths, and limited greenery, representing a typical urban street scene with higher condensation sink and black carbon concentrations than the urban background.

S1.5.3. Juan Carlos I research base, Antarctica

Measurements were conducted at the Juan Carlos I Spanish research station (62° 39' 36" S, 60° 23' 24" W) on the south coast of Livingston Island in the South Shetland Islands between February 12 and March 13, 2019. The Juan Carlos I station is characterized by pristine airmasses flowing over ocean, sea ice, and continental and coastal Antarctica.

S1.5.4. Mace Head research station, Ireland

The measurements at the Mace Head atmospheric research station on the west coast of Ireland (53°19’ N, 9°54’W) were done in August - October 2013. Mace Head is known for the strong tidal changes and presence of iodine emitting macroalgae beds consisting of e.g. kelps such as different *Laminaria* species[8] and other brown algae such as *Ascophyllum nodosum* and *Fucus vesiculosus*[9]. Mace Head represents of typical North Atlantic rocky shores in terms of the macroalgae exposure times.

The concentration of sulfuric acid was calculated by considering signal intensity of $HNO_3*HSO_4^-$ (159.9557 Th) only. The signal peak corresponding to HSO_4^- (96.9601 Th) was overlapping with the peak of $ClCH_2O_3^-$ (96.9698 Th) and was not properly resolved (Figure

S17 B). This might be due to the resolution of the mass spectrometer. Thus, we have not included HSO_4^- signal in calculating the H_2SO_4 concentration as well as calibration coefficient.

S1.5.5. Maïdo Observatory, Réunion island

All the measurements were performed at the framework of OCTAVE 2018 campaign at the Maïdo observatory located on the island of Réunion, in the Indian Ocean (21.080° S, 55.383° E; 2150). The data were collected between 7 March and 8 May 2018 and specific attention has been given on the timeframe between 28 April to 4 May, during which the volcanic eruption plume of Piton de la Fournaise, situated ~ 39 km from Maïdo in the south-eastern region of the island, was detected at the station. Detailed information about the instrumental set up is given in Rose et al.[10] Metrological parameters including relative humidity (RH; Vaisala Weather Transmitter WXT510) were measured with 3 s time resolution.

Table S2. NO_3^--CIMS inlet flow (l min^{-1}) and calibration coefficient ($C_{H_2SO_4}$; molecules cm^{-3}) at different measurement locations.

Measurement site	Inlet flow of the NO_3^- CIMS (liters min^{-1})	$C_{H_2SO_4}$ (molecules cm^{-3})
Urban Background, Leipzig, Germany	8	1.07×10^{9} [a]
Roadside, Leipzig, Germany	10	2.05×10^{9} [a]
Juan Carlos I research base, Antarctica	15	1×10^{10} [b]
Mace Head research station, Ireland	10	1.3×10^{9} [b]
Maïdo Observatory, Réunion island	10	1.7×10^{10} [b]

[a] determined by using the updated methodology of Mettke et al.[7]
[b] determined by the approach detailed in Kürten et al.[11]

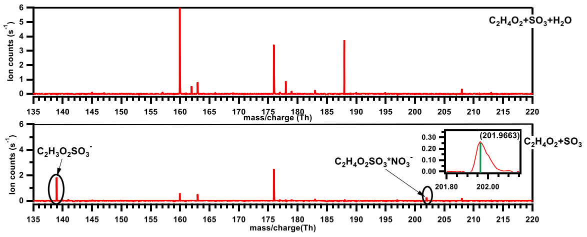

Figure S2. Mass spectra for the reaction of AA with SO_3 in the flow reactor. The mass spectrum in the presence and absence of water is shown in the upper and lower panel respectively.

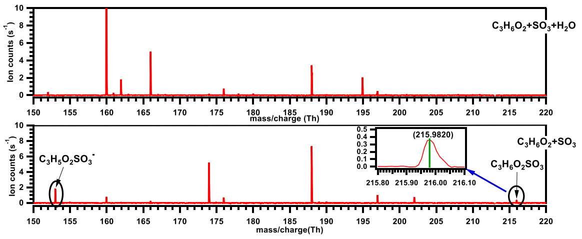

Figure S3. Mass spectra for the reaction of PA with SO_3. The mass spectrum in the presence and absence of water is shown in the upper and lower panel respectively.

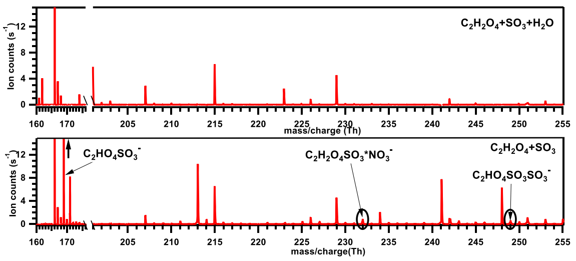

Figure S4. Mass spectra for the reaction of OA with SO_3. The mass spectrum in the presence and absence of water is shown in the upper and lower panel respectively.

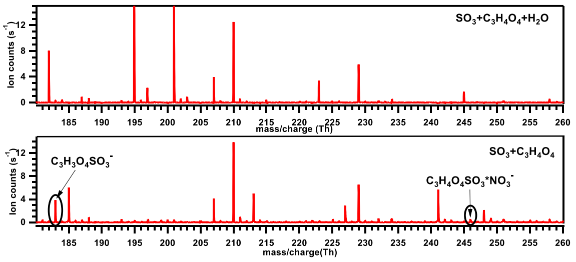

Figure S5. Mass spectra for the reaction of MA with SO_3. The mass spectrum in the presence and absence of water is shown in the upper and lower panel respectively.

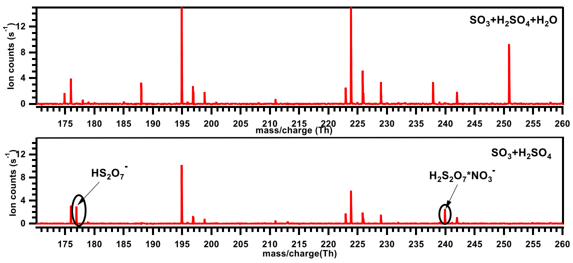

Figure S6. Mass spectra for the reaction of SA with SO_3. The mass spectrum in the presence and absence of water is shown in the upper and lower panel respectively.

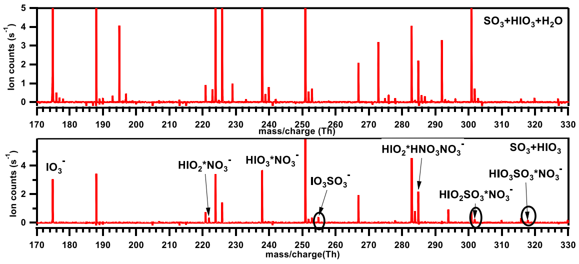

Figure S7. Mass spectra for the reaction of IA with SO_3. The mass spectrum in the presence and absence of water is shown in the upper and lower panel respectively.

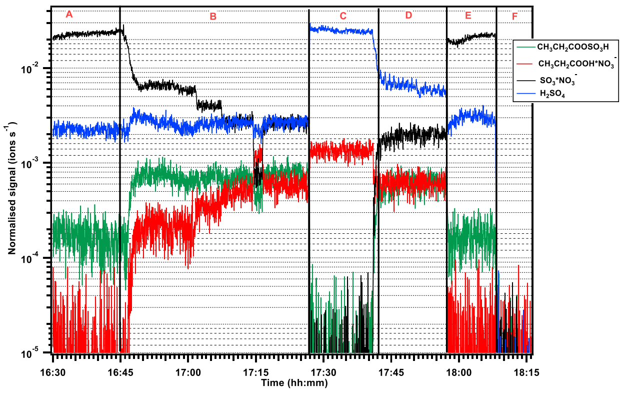

Figure S8. Normalized time series plot obtained during the reaction of PA with SO_3. A: injection of SO_2 and OH radical (formation of SO_3); C: injection of PA; D: injection of water in the reaction system; E: stopped injection of water; F: stopped injection of PA; G: stopped ozone. The normalized signal of $CH_3CH_2COOSO_3H$ and H_2SO_4 is represented as $(S_{CH_3CH_2COOSO_3^-} + S_{CH_3CH_2COOSO_3H*NO_3^-})$ and $(S_{HSO_4^-} + S_{H_2SO_4*NO_3^-})$ respectively.

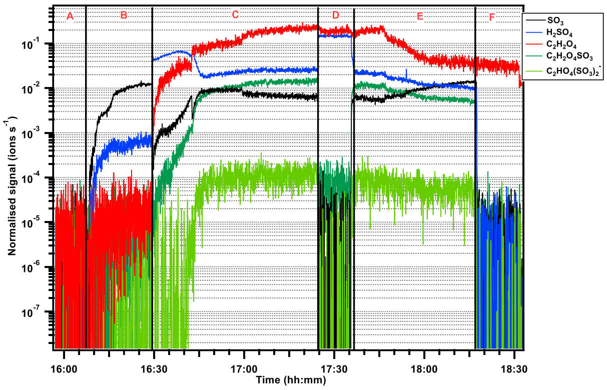

Figure S9: Normalized time series plot obtained during the reaction of OA with SO_3. A: Background; B: injection of SO_2 and OH radical (formation of SO_3); C: injection of OA; D: injection of water in the reaction system; E: stopped injection of water; F: stopped ozone. The normalized signal of $C_2H_2O_4$, $C_2H_2O_4SO_3$ and H_2SO_4 is represented as $\left(S_{C_2HO_4^-} + S_{C_2H_2O_4*NO_3^-}\right)$, $\left(S_{C_2HO_4SO_3^-} + S_{C_2H_2O_4SO_3*NO_3^-}\right)$ and $\left(S_{HSO_4^-} + S_{H_2SO_4*NO_3^-}\right)$ respectively.

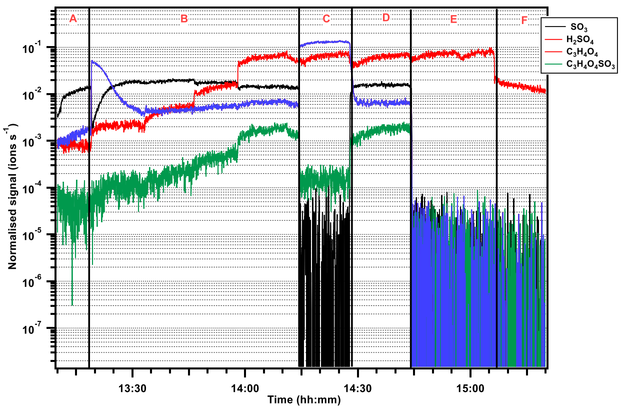

Figure S10. Normalized time series plot obtained during the reaction of MA with SO_3. A: injection of SO_2 and OH radical (formation of SO_3); B: injection of MA; C: injection of water in the reaction system; D: stopped injection of water; E: stopped O_3; F: stopped MA. The normalized signal of $C_3H_4O_4$, $C_3H_4O_4SO_3$ and H_2SO_4 is represented as $\left(S_{C_3H_3O_4^-}+S_{C_3H_4O_4*NO_3^-}\right)$, $\left(S_{C_3H_3O_4SO_3^-}+S_{C_3H_4O_4SO_3*NO_3^-}\right)$ and $\left(S_{HSO_4^-}+S_{H_2SO_4*NO_3^-}\right)$ respectively.

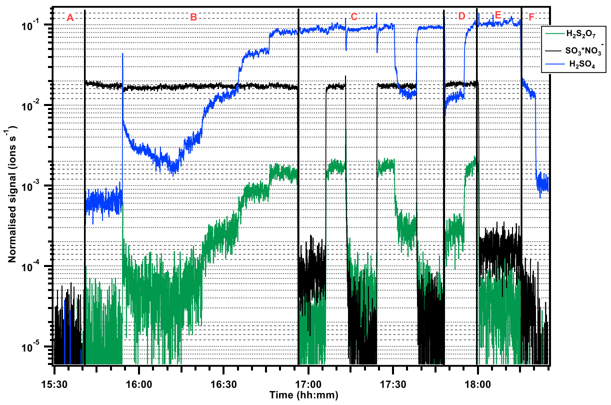

Figure S11. Normalized time series plot obtained during the reaction of SA with SO_3. A: Background; B: injection of SO_2 and OH radical (formation of SO_3); C: injection of SA; D: injection of water in the reaction system and decreasing the flow of SA; D: stopped injection of water; E: stopped ozone; F: stopped injection of SA. The normalized signal of $H_2S_2O_7$ and H_2SO_4 is represented as $(S_{HS_2O_7^-} + S_{HNO_3*HS_2O_7^-})$ and $(S_{HSO_4^-} + S_{H_2SO_4*NO_3^-})$ respectively.

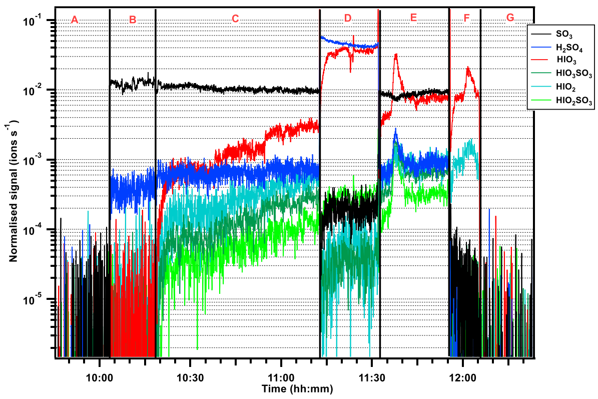

Figure S12. Normalized time series plot obtained during the reaction of IA with SO_3. A: background; A: injection of SO_2 and OH radical (formation of SO_3); C: injection of I_2 vapors; D: injection of water in the reaction system; E: stopped injection of water; F: stopped the flow of SO_2; G: stopped ozone. The normalized signal of HIO_3, HIO_3SO_3 and H_2SO_4 is represented as $\left(S_{IO_3^-} + S_{HIO_3*NO_3^-}\right)$, $\left(S_{IO_3SO_3^-} + S_{HIO_3SO_3*NO_3^-}\right)$ and $(S_{HSO_4^-} + S_{H_2SO_4*NO_3^-})$ respectively.

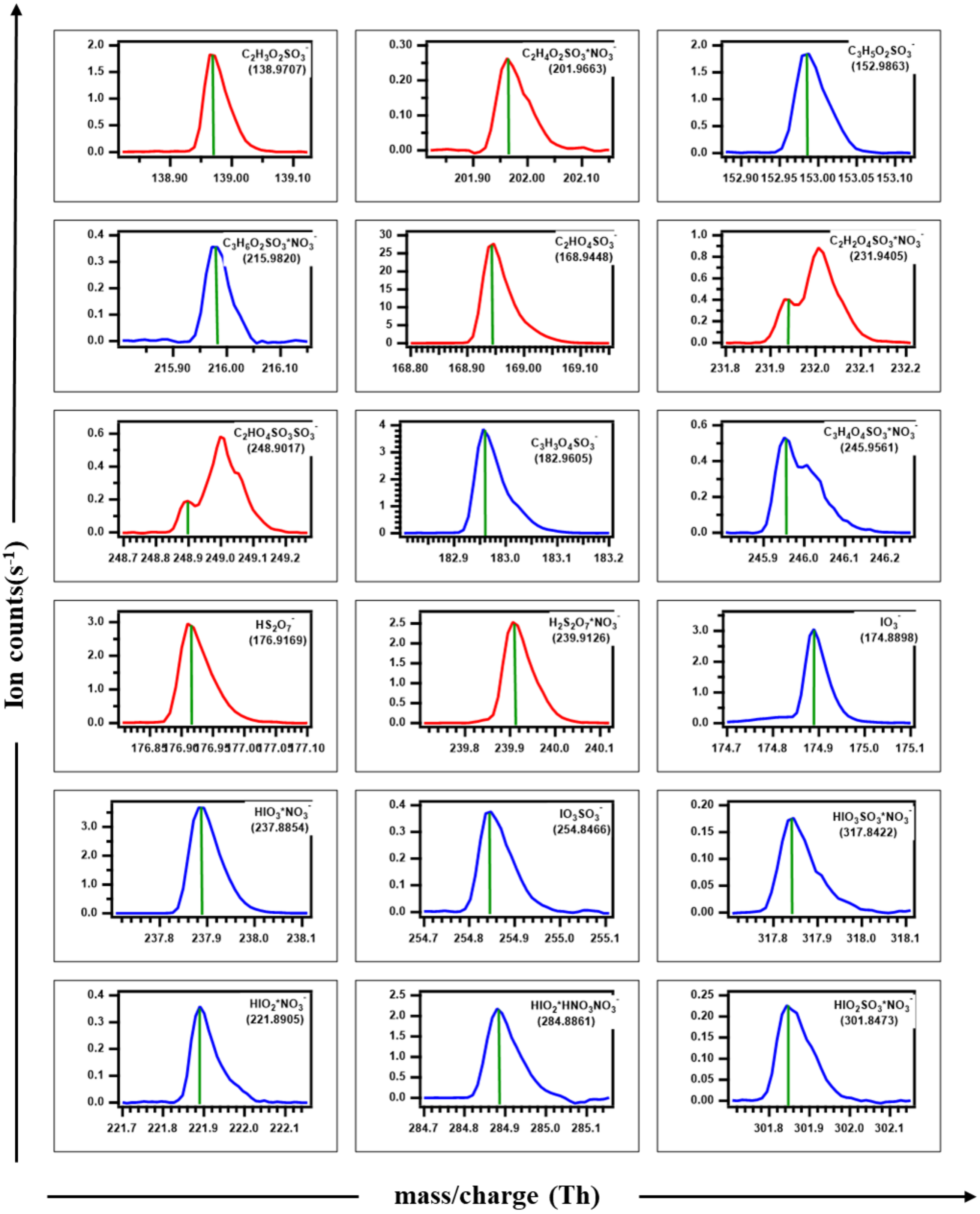

Figure S13. High-resolution peak fitting of the product signals formed in all the studied reactions.

Table S3. Second SO_3 addition to di-carboxylic acids. Energies of the stationary points are calculated at the RHF-RCCSD(T)-F12a/VDZ-F12//ωB97X-D/aug-cc-PV(T+d)Z level of theory.

Molecule + SO_3	ΔG kcal/mol (relative to separated reactants)		
	RC	TS	P
$OASO_3H$	+0.5	+4.2	-1.4
$MASO_3H$	+0.6	+5.1	-1.6[a]

[a] Energy calculated at the ωB97X-D/aug-cc-pV(T+d)Z level of theory.

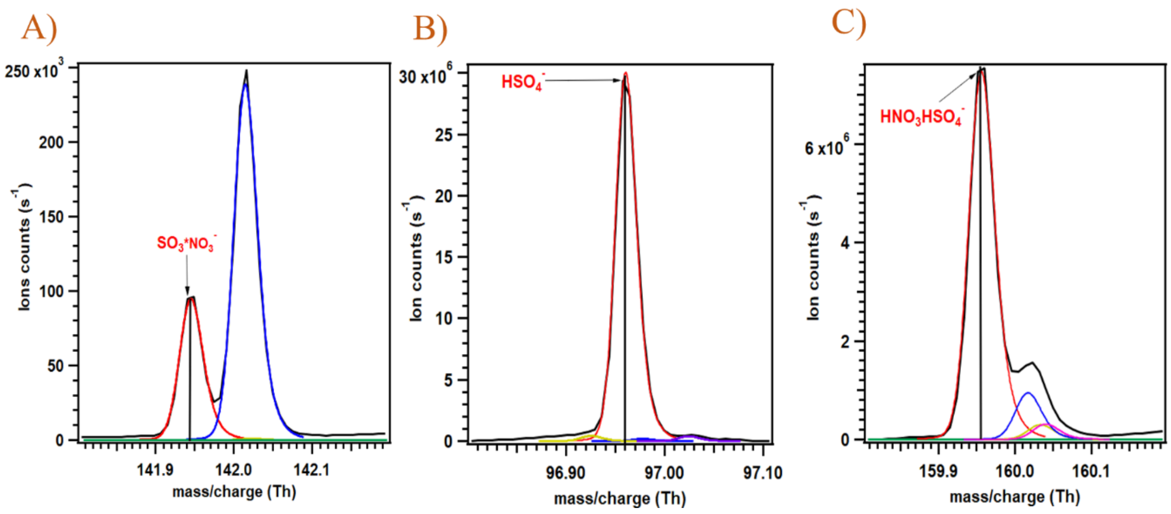

Figure S14. High-resolution peak fitting of A) $SO_3*NO_3^-$ and B) HSO_4^- and C) $HNO_3*HSO_4^-$ measured at the urban roadside measurement site in Leipzig, Germany. The black and red color trace represents the raw spectrum and fitted peak of the ion of interest respectively.

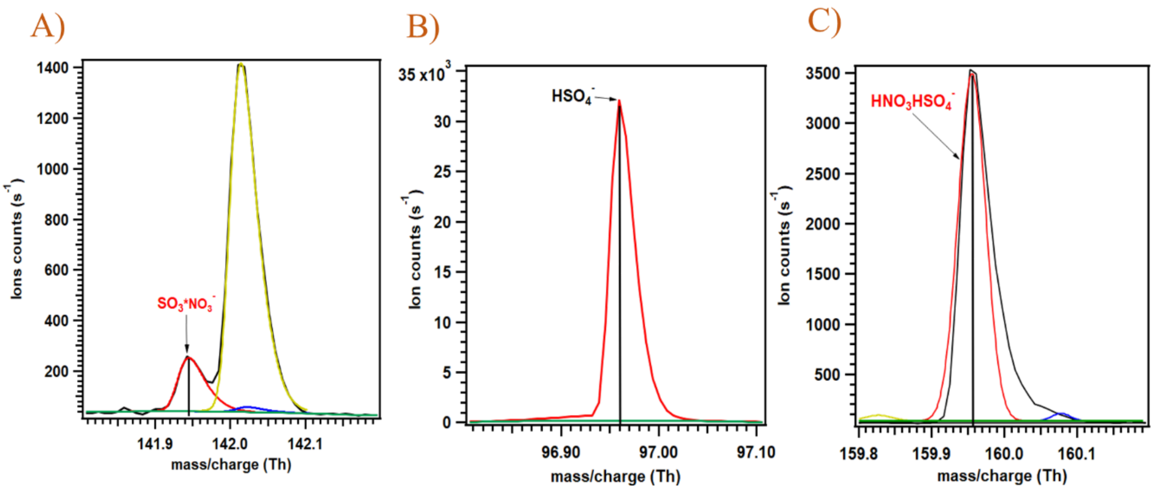

Figure S15. High-resolution peak fitting of A) SO_3*NO_3^- and B) HSO_4^- measured at the urban background measurement site in TROPOS, Leipzig, Germany. The black and red color trace represents the raw spectrum and fitted peak of the ion of interest respectively.

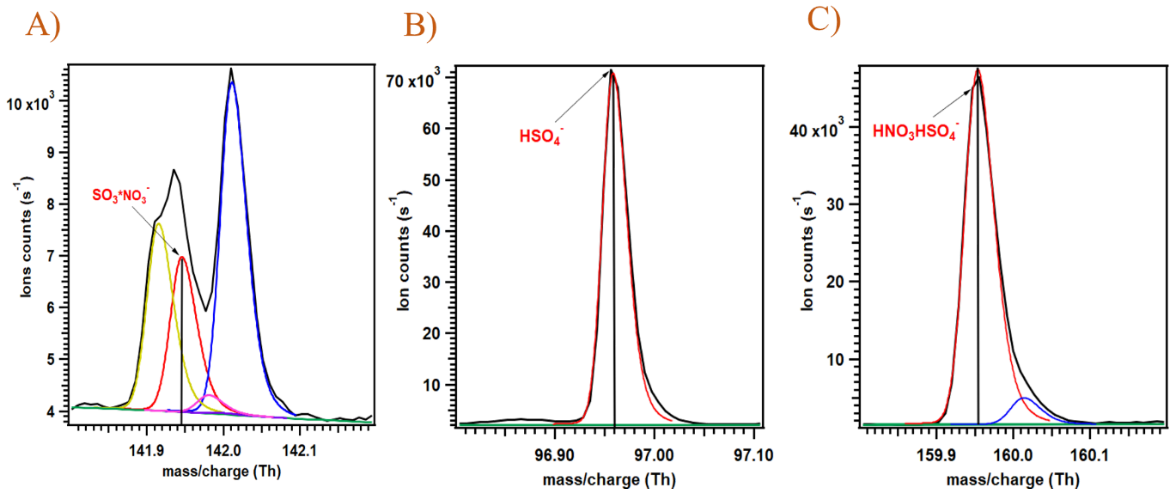

Figure S16. High-resolution peak fitting of A) SO_3*NO_3^- and B) HSO_4^- measured at the research station Juan Carlos I, Antarctic Peninsula. The black and red color trace represents the raw spectrum and fitted peak of the ion of interest respectively.

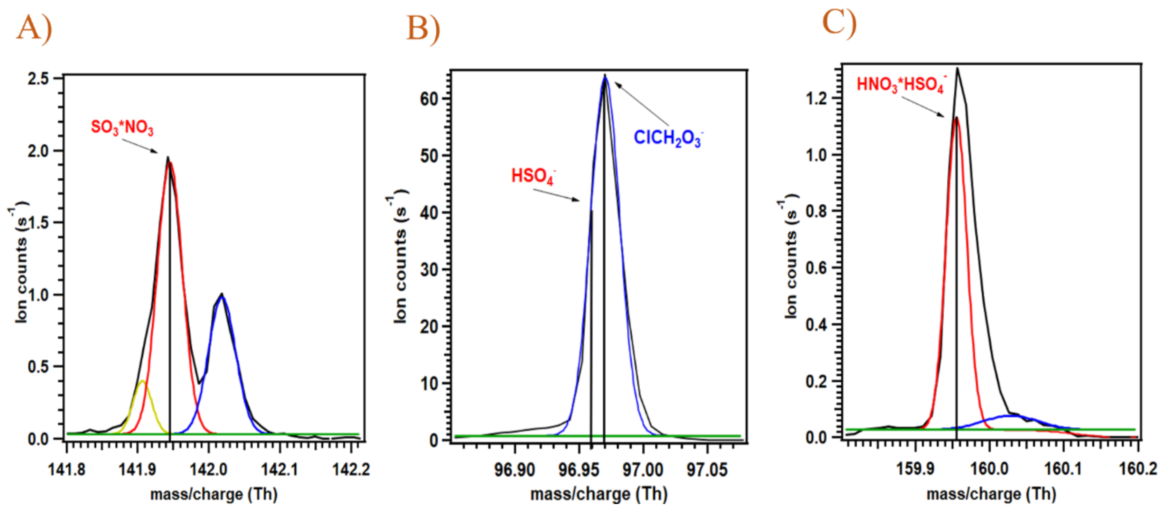

Figure S17. High-resolution peak fitting of A) SO_3*NO_3^-, B) HSO_4^- and C) HNO_3*HSO_4^- measured at the Mace Head research station, Ireland. The black and red color trace represents the raw spectrum and fitted peak of the ion of interest respectively.

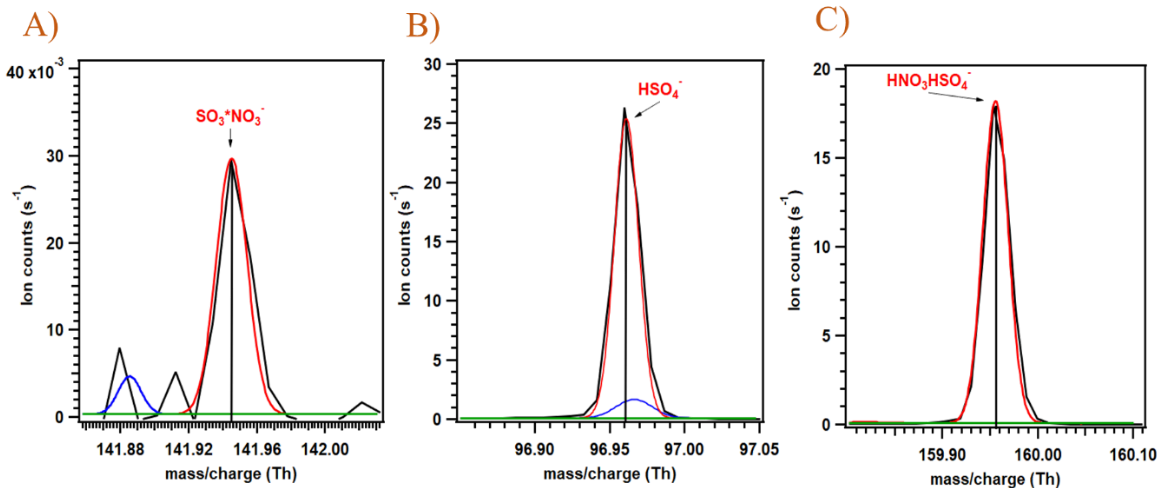

Figure S18. High-resolution peak fitting of A) SO_3*NO_3^-, B) HSO_4^- and C) HNO_3*HSO_4^- measured at Maïdo Observatory, Réunion island. The black and red color trace represents the raw spectrum and fitted peak of the ion of interest respectively.

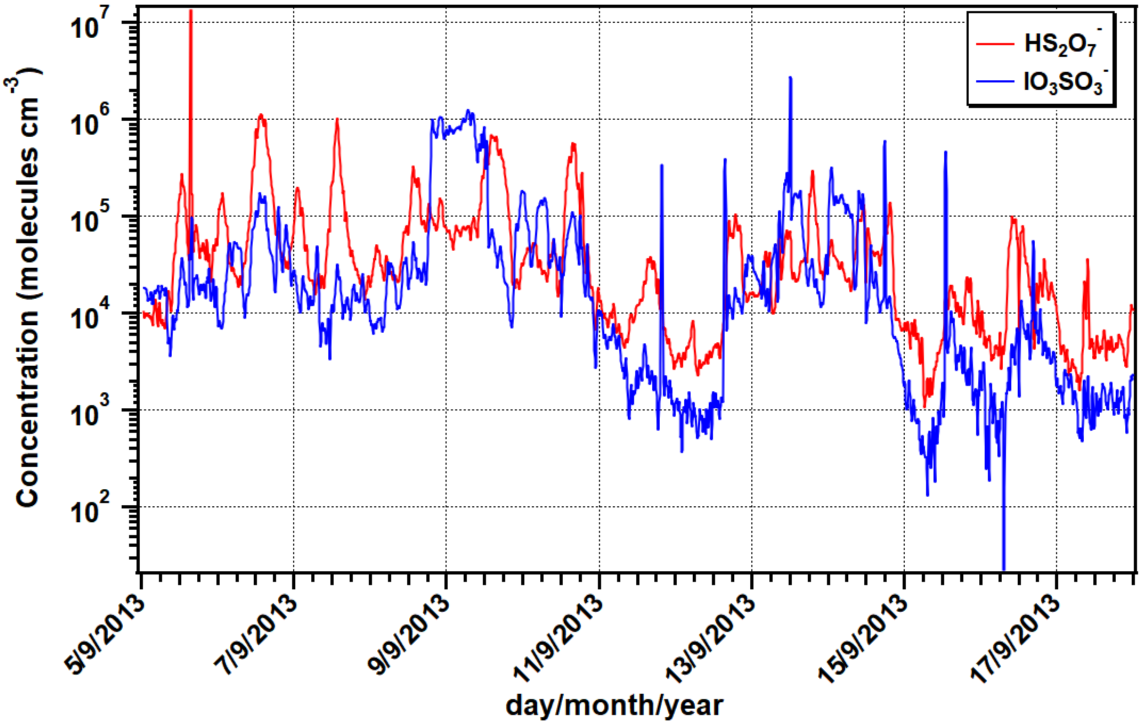

Figure S19. Concentration time series plot for disulfuric acid ($HS_2O_7^-$) and iodic sulfuric anhydride ($IO_3SO_3^-$) measured at the Mace Head research station, Ireland.

Table S4. Atmospheric lifetimes (s) of SO_3 with respect to its bimolecular reaction with water dimer and studied acids in the temperature range of 275 – 320K.

T (K)	τ_{SO_3} (s)							
	RH=20%[a]	RH=40% [a]	AA[b]	PA[b]	OA[c]	MA[c]	SA[c]	IA[c]
275	8.45×10^{-4}	2.11×10^{-4}	1.59×10^{-2}	1.39×10^{-2}	8.24×10^{2}	1.33×10^{3}	2.98×10^{2}	1.58×10^{2}
290	1.72×10^{-4}	4.28×10^{-5}	1.66×10^{-2}	1.43×10^{-2}	9.63×10^{2}	1.62×10^{3}	3.45×10^{2}	1.78×10^{2}
298	7.44×10^{-5}	1.86×10^{-5}	1.69×10^{-2}	1.45×10^{-2}	1.04×10^{3}	1.78×10^{3}	3.71×10^{2}	1.88×10^{2}
310	2.48×10^{-5}	6.21×10^{-6}	1.74×10^{-2}	1.47×10^{-2}	1.16×10^{3}	2.04×10^{3}	4.10×10^{2}	2.04×10^{2}
320	1.09×10^{-5}	2.74×10^{-6}	1.78×10^{-2}	1.50×10^{-2}	1.26×10^{3}	2.26×10^{3}	4.43×10^{2}	2.18×10^{2}

[a] The concentration of water dimer was obtained from Anglada et al.[12]. [b]The average concentration for acetic acid (AA) and propionic acid (PA) used in the calculation is 4×10^{11} molecules cm $^{-3}$. [c]The average concentration for oxalic acid (OA), malonic acid (MA) sulfuric acid (SA) and iodic acid (IA) used in the calculation is 1×10^{8} molecules cm $^{-3}$.

References

(1) Junninen, H.; Ehn, M.; Petäjä, T.; Luosujärvi, L.; Kotiaho, T.; Kostiainen, R.; Rohner, U.; Gonin, M.; Fuhrer, K.; Kulmala, M.; Worsnop, D. R. A high-resolution mass spectrometer to measure atmospheric ion composition, *Atmos. Meas. Tech.* **2010**, *3*, 1039-1053.

(2) Eisele, F. L. & Tanner, D. J. Measurement of the gas phase concentration of H2SO4 and methane sulfonic acid and estimates of H2SO4 production and loss in the atmosphere. *J. Geophys. Res.* **1993**, *98*, 9001–9010.

(3) Hyttinen, N.; Kupiainen-Määttä, O.; Rissanen, M. P.; Muuronen, M.; Ehn, M.; Kurtén, T. Modeling the Charging of Highly Oxidized Cyclohexene Ozonolysis Products Using Nitrate-Based Chemical Ionization, *J. Phys. Chem. A* **2015**, *119*, 6339–6345.

(4) Plane, J. M. C.; Joseph, D. M.; Allan, B. J., Ashworth, S. H.; Francisco, J. S. An experimental and theoretical study of the reactions OIO + NO and OIO + OH. *J. Phys. Chem. A* **2006**, *110*, 93–100.

(5) Finkenzeller, H.; Iyer, S.; He, X.-C.; Simon, M.; Koenig, T. K.; Lee, C. F.; Valiev, R.; Hofbauer, V.; Amorim, A.; Baalbaki, R.; Baccarini, A.; Beck, L.; Bell, D. M.; Caudillo, L.; Chen, D.; Chiu, R.; Chu, B.; Dada, L.; Duplissy, J.; Heinritzi, M.; Kemppainen, D.; Kim, C.; Krechmer, J.; Kürten, A.; Kvashnin, A.; Lamkaddam, H.; Lee, C. P.; Lehtipalo, K.; Li, Z.; Makhmutov, V.; Manninen, H. E.; Marie, G.; Marten, R.; Mauldin, R. L.; Mentler, B.; Müller, T.; Petäjä, T.; Philippov, M.; Ranjithkumar, A.; Rörup, B.; Shen, J.; Stolzenburg, D.; Tauber, C.; Tham, Y. J.; Tomé, A.; Vazquez-Pufleau, M.; Wagner, A. C.; Wang, D. S.; Wang, M.; Wang, Y.; Weber, S. K.; Nie, W.; Wu, Y.; Xiao, M.; Ye, Q.; Zauner-Wieczorek, M.; Hansel, A.; Baltensperger, U.; Brioude, J.; Curtius, J.; Donahue, N. M.; Haddad, I. E.; Flagan, R. C.; Kulmala, M.; Kirkby, J.; Sipilä, M.; Worsnop, D. R.; Kurten, T.; Rissanen, M.; Volkamer, R. The gas-phase formation mechanism of iodic acid as an atmospheric aerosol source. *Nat. Chem.* **2023***, 15,* 129–135.

(6) He, X.-C.; Simon, M.; Iyer, S.; Xie, H.,-B.; Rörup, B.; Shen, J.; Finkenzeller, H.; Stolzenburg, D.; Zhang, R.; Baccarini, A.; Tham, Y. J.; Wang, M.; Amanatidis, S.; Piedehierro, A. A.; Amorim, A.; Baalbaki, R.; Brasseur, Z.; Caudillo, L.; Chu, B.; Dada, L.; Duplissy, J.; Haddad, I. E.; Flagan, R. C.; Granzin, M.; Hansel, A.; Heinritzi, M.; Hofbauer, V.; Jokinen, T.; Kemppainen, D.; Kong, W.; Krechmer, J.; Kürten, A.; Lamkaddam, H.; Lopez, B.; Ma, F.; Mahfouz, N, G, A.; Makhmutov, V.; Manninen, H. E.; Marie, G.; Marten, R.; Massabò, D.;

Mauldin, R. L.; Mentler, B.; Onnela, A.; Petäjä, T.; Pfeifer, J.; Philippov, M.; Ranjithkumar, A.; Rissanen, M. P.; Schobesberger, S.; Scholz, W.; Schulze, B.; Surdu, M.; Thakur, R, C.; Tomé, A.; Wagner, A. C.; Wang, D.; Wang, Y.; Weber, S. K.; Welti, A.; Winkler, P. M.; Wieczorek, M. Z.; Baltensperger, U.; Curtius, J.; Kurtén, T.; Worsnop, D. R.; Volkamer, R.; Lehtipalo, K.; Kirkby, J.; Donahue, N. M.; Sipilä, M.; Kulmala, M. Iodine oxoacids enhance nucleation of sulfuric acid particles in the atmosphere. *Science* **2023***, 382,*1308-1314.

(7) Mettke, P., Brüggemann, M., Mutzel, A., Gräfe, R. and Herrmann, H., 2023. Secondary Organic Aerosol (SOA) through Uptake of Isoprene Hydroxy Hydroperoxides (ISOPOOH) and its Oxidation Products. *ACS Earth and Space Chemistry*, *7*(5), pp.1025-1037.

(8) McFiggans, G.; Coe, H.; Burgess, R.; Allan, J.; Cubison, M.; Alfarra, M.R.; Saunders, R.; Saiz-Lopez, A.; Plane, J.M.C.; Wevill, D.; Carpenter, L. Direct evidence for coastal iodine particles from Laminaria macroalgae: linkage to emissions of molecular iodine. *Atmos. Chem. Phys*. **2004**, *4*, 701–713.

(9) Huang, R. J.; Thorenz, U.R.; Kundel, M.; Venables, D.S.; Ceburnis, D.; Ho, K.F.; Chen, J.; Vogel, A.L.; Küpper, F.C.; Smyth, P.P.A.; Nitschke, U. The seaweeds Fucus vesiculosus and Ascophyllum nodosum are significant contributors to coastal iodine emissions. *Atmos. Chem. Phys.* **2013**, *13*, 5255-5264.

(10) Rose, C.; Foucart, B.; Picard, D.; Colomb, A.; Metzger, J.-M.; Tulet, P.; Sellegri, K. New particle formation in the volcanic eruption plume of the Piton de la Fournaise: specific features from a long-term dataset, *Atmos. Chem. Phys.* **2019**, *19*, 13243–13265.

(11) Kürten, A.; Rondo, L.; Ehrhart, S.; Curtius, J. Calibration of a chemical ionization mass spectrometer for the measurement of gaseous sulfuric acid. *J. Phys. Chem. A* **2012**, *116,* 6375-6386.

(12) Anglada, J. M.; Hoffman, G. J.; Slipchenko, L. V.; M.Costa, M. M.; Ruiz-López, M. F.; Francisco, J. S. Atmospheric significance of water clusters and ozone-water complexes. *J. Phys. Chem. A* **2013**, *117*, 10381– 10396.
